# Similarity of gaze patterns across physical and virtual versions of an installation artwork

**DOI:** 10.1038/s41598-021-91904-x

**Published:** 2021-09-23

**Authors:** Doga Gulhan, Szonya Durant, Johannes M. Zanker

**Affiliations:** Department of Psychology, Royal Holloway, University of London, Surrey, UK

**Keywords:** Human behaviour, Visual system

## Abstract

An experiment was conducted to compare museum visitors’ gaze patterns using mobile eye-trackers, whilst they were engaging with a physical and a virtual reality (VR) installation of Piet Mondrian’s Neo-plasticist room design. Visitors’ eye movements produced approximately 25,000 fixations and were analysed using linear mixed-effects models. Absolute and area-normalized dwell time analyses yielded mostly non-significant main effects of the environment, indicating similarity of visual exploration patterns between physical and VR settings. One major difference observed was the decrease of average fixation duration in VR, where visitors tended to more rapidly switch focus in this environment with shorter bursts of attentional focus. The experiment demonstrated the ability to compare gaze data between physical and virtual environments as a proxy to measure the similarity of aesthetic experience. Similarity of viewing patterns along with questionnaire results suggested that virtual galleries can be treated as ecologically valid environments that are parallel to physical art galleries.

## Introduction

Empirical aesthetics emerged in the late nineteenth century^1,2^, and was roughly contemporaneous with the foundation of experimental psychology and psychophysics. Following on, pioneering eye-tracking research^3,4^ was also asking questions on how observers engage with artworks visually, whilst another line of inquiry was aiming to capture the external world via photography. Now, half a century after the earliest computer-generated 3D movies^5^ and VR headsets^6^, recent developments in computational power and VR eye-tracking^7^ allow researchers to conduct experiments in accurate recreations of 3D environments. This includes our work presented here, which made use of a unique opportunity to compare visual exploration of a single installation artwork alongside its VR reconstruction in a museum space, leading the way to evaluating the validity of virtual and online arts experience.

In line with psychophysical approaches, visual artworks have been generally treated as controllable or categorizable stimuli^8,9^. Similarly, and on a more theoretical level, approaches to conceptualise aesthetics “from below” on the basis of visual information processing assume that perception of artworks relies mostly on bottom-up processing with minimum influence from top-down processing. Generally, bottom-up (data-based) processing frames visual perception as a stimulus-driven and direct phenomenon, whereas top-down (knowledge-based) processing underlines the influence of past experience and prior knowledge on visual perception, and describes it as a more indirect, inference-making process^10,11^, although this arguably imprecise dichotomy calls for further conceptual refinements^12^**.** Following criticism of some reductionist approaches to aesthetics^13^ and evidence from less restrictive experiments, detailed models of aesthetic experience were suggested^14–17^, often embracing both the universality of aesthetic experience as a result of low level visual neural dynamics and the diversity of aesthetic experience as a result of contextual and personal factors^18^. A similar inclusive viewpoint argues for influences of both bottom-up and top-down processing on (overt) visual attention and attention-related tasks^19,20^, which can be linked to eye movements in general, and fixation-related metrics in particular.

Although aesthetic experience, as with other related concepts, is a highly debatable topic in itself by both theorists and experimentalist^21–24^, and prone to circular definitions, at its simplest, it refers to a particular state of mind whilst engaging with an artwork, a context-dependent spatiotemporal episode of the conscious experience. The concept of attention can be linked to visual perception and more particularly to the visual aesthetic experience^25,26^, since it can be described as a mechanism that shapes (e.g. selects, concentrates, distributes) the information received from the scene, although linking theoretical work and empirical findings related to attention into a coherent description is an ongoing challenge^27^. Assuming aesthetic experience to be a set of highly complex cognitive-emotional processes involving attentional mechanisms, eye movements can be seen as a reflection of both underlying bottom-up and top-down processes.

Until recently, the only feasible way of implementing eye-tracking in a study was by restricting it to laboratory environments, where generally the only stimulus option was the reproduction of artworks instead of originals. In line with the development of mobile eye trackers, a paradigm-shift for empirical aesthetic research was stepping outside of the well-controlled laboratory environments into the real world where observers engage with works of art in their original forms^28^. In a previous pilot experiment, for example, we investigated the eye movements of gallery visitors whilst they were engaging with a room-scale installation^29^. This installation was later recreated virtually with a set of variations based on the topological properties and observed gaze patterns, and these variations were used in an online 2D view eye-tracking experiment. To further illustrate this research potential, researchers have investigated (1) interaction between gaze patterns and abstract paintings in a gallery^30^ and potential implementation of scan path analysis using support-vector machine algorithms to classify paintings based on fixation sequences^31^, (2) use of mobile eye-tracking analysis on abstract and representational paintings in a museum^32^, (3) effects of bottom-up factors (as indexed by saliency maps derived from paintings) and top-down factors (as manipulated by the information about paintings provided to the participants, who were allowed to view the same paintings again) between children and adults, whilst viewing Van Gogh paintings^33^, (4) interaction between speaking and fixation patterns and various gaze metrics^34^, (5) difference in exploration strategies among wheelchair and non-chair users in museums^35^, (6) amount of attentional shift between museum content itself and a supplementary tablet containing information on that content^36^, (7) whether fixation duration can predict aesthetic choice^37^—among others. One commonality across these diverse studies is their emphasis on the necessity of fieldwork in empirical aesthetics, aiming to measure aesthetic experience and judgments in genuine settings.

Presenting arts online and more recently virtually was a huge step forward for accessibility of cultural heritage. Although VR has been used previously in pioneering works^38^ and research^39^, there is currently a growing interest in both consumer-grade and research-grade VR solutions. One particular reasoning behind this interest seems to be the experimental research potential to employ freely moving participants in virtual environments^40–42^. Also, the accessibility to modelling software and game engines provides ease and widely accessible tools to create novel immersive environments^43–45^. Additionally, following the development of eye movement analysis in 3D space collected from digital simulated environments^46,47^, the recent emergence of VR headsets capable of eye-tracking offers a completely new opportunity to step beyond the conventional lab and into the in-situ context. A relatively unexplored area with emerging experimental design guidelines as well as some ethical concerns^48,49^, nevertheless VR holds exciting promise for empirical aesthetic research. There is some previous research comparing museum and laboratory settings as well as original and reproduction artworks^50^, and similarly, investigating preference towards types of substituted representations of artworks^51^, or targeting emotional experience using mobile EEG to develop a classifier based on the data recordings from a real and virtual museum^52^. In line with recent experimental results underlining the observed contextual differences (particularly between lab-based and real-world conditions), aesthetics research in laboratories resembling the genuine contexts of aesthetic experience as much as possible was proposed^53^. However, direct comparative research between the art galleries and arguably their closest proxy, immersive environments, (particularly for 3D arts and based on eye-tracking) is still missing. As the VR environment develops into a valid and comparable setting to physical galleries and museums, a direct comparison between VR and in-situ environments seems to be crucial to enable future use of VR. If such research suggests similarity between the two settings, then immersive environments can be treated as both highly controllable and ecologically valid research settings.

Here we focus on the work of Piet Mondrian, whose abstract paintings are prominent examples of the De Stijl art movement. In most of his late works, Mondrian radically restricted compositional features of artworks following the art movement of Neoplasticism, by using only horizontal and vertical lines and three primary colours red, blue, and yellow, along with black, white, and grey. This abstraction epitomising purity and sparseness of lines and colours lends itself to straight-forward mathematical descriptions to aid quantitative approaches. In this sense, reproduction of Mondrian paintings and quasi-Mondrians as manipulated versions of originals have been used as stimuli for empirical aesthetics, arguably due to the artist’s historical significance as well as the low-level compositional features, offering clear and easily modifiable geometric structures as experimental stimuli. For example, researchers investigated whether computer-generated synthetic Mondrians were preferred more compared to originals^54,55^, whether original or rotated, oblique orientations of Mondrian’s paintings were preferred and whether eye-movement patterns were similar across orientation conditions^56^, and whether aesthetic preference towards Mondrian paintings was correlated with measured pupil size of participants^57^. Incorporation of other variables in relation to Mondrian’s work has also been a prominent research theme, such as asking whether liking of original Mondrians was mediated by personality factors like openness to experience^58^. Recently, a distinct example of Mondrian’s work, a room design proposal commissioned by Ida Bienert in the early twentieth century but never realised^59^ has drawn attention from researchers, along with an art-historic curiosity. Using variations of scale physical and digital models, it was argued that the room-scale artwork proposal conflicted with strict neoplasticist ideals, because of perspective distortions in retinal projections, which are exacerbated by changes of viewpoints in the room^60^. Following on from that, our test case mainly aims to measure observers’ visual exploration as indexed by eye-trackers inside 1:1 scale physical and virtual versions of this particular design proposal. In terms of art research, our approach can be seen both as a behavioural experiment in a physical gallery, and as a comparative study aimed to investigate whether a virtual installation would be a suitable proxy for a physical installation.

The main aim of the present study was to compare observers’ gaze patterns (as constituents of the aesthetic experience) within an art installation in physical and virtual instantiations. The physical installation created by the artist Heimo Zobernig, and the VR reconstruction developed by our team were temporarily exhibited in the Albertinum Museum in Dresden, Germany. Having a full day of access to a flagship exhibition on historical milestones of abstract art in an internationally renowned museum^61^ to quantitatively analyse looking behaviour in a live gallery setting and to assess the similarity between physical and VR contexts was a unique opportunity for us. In this case study, we collected both implicit measures as ocular responses using a mobile eye-tracker for the physical installation and using a VR eye-tracker for a virtual reconstruction, and supplementary explicit measures as questionnaire responses. Specific ocular responses related to fixations, such as dwell time and fixation count, are usually associated with overt attention, visual attention guidance, and other related (unconscious) cognitive processes^62^. On the other hand, questionnaire responses as a part of psychological testing were considered to reflect decision making and other (conscious) cognitive processes^63^. As a result, to the best of our knowledge, we present the first direct comparison of quantitative measures capturing core aspects of visual aesthetic experience of an installation artwork in both physical and virtual embodiment in its museum context. Given the strong topological similarity between physical and VR installations, we expect very similar visual exploration behaviours between the two contexts. In this sense, the non-directional alternative hypothesis can be formulated such that the visual exploration patterns as indexed by absolute and area-normalized fixation duration regarding sets of area of interests (AOIs) during the viewing of a static abstract installation between in-situ and VR condition are different, whereas the null hypothesis as the default state can be formulated that there is no difference between the two contexts.

## Methods

### Participants

Museum visitors were approached at the exhibition entrance and invited to take part using opportunity sampling during regular visiting hours. They took part in the study voluntarily. All participants provided written informed consent prior to the experiment. Thirty-one museum visitors (21 females, 9 males, *M*_*Age*_ = 49.23 years, *SD*_*Age*_ = 18.25 years, *R*_*Age*_ = 20–79 years) participated in the study. All participants reported having normal or corrected-to-normal vision, in the sense that they viewed both stimuli in the same conditions as if they were viewing other artworks of the exhibition. Participants could use their contact lenses for both settings, or wear their glasses in the VR headset and a corrective lens was added to the wearable eye tracker whenever needed. Although no explicit vision status measure such as a visual acuity or contrast sensitivity test was implemented; a screening questionnaire comprising eleven items was provided, aiming to link any unusual eye-tracking data (such as calibration failure or frequent lack of fixation detection) to the vision condition (such as recent laser surgery), and potentially to exclude the participant data (see Supplementary Fig. S1 for the screening form and exit questionnaire, English version). All experimental protocols were approved by the Royal Holloway, University of London Research Ethics Committee. All methods were performed in accordance with the ethical guidelines and regulations of the Declaration of Helsinki.

### Stimuli and materials

**Figure 1:**
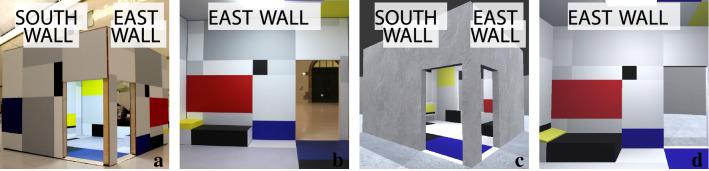
Physical and virtual versions of Mondrian’s room design. (**a**) The exterior and (**b**) the interior photographs of the physical installation created by artist Heimo Zobernig. Similarly, (**c**) the exterior and (**d**) the interior views of VR reconstruction, developed by our research team. In the physical installation, one of the artistic decisions of Heimo Zobernig was to extrude the interior patterns onto exterior surfaces of the room, whereas our VR reconstruction had a homogeneous grey texture for the exterior surfaces. Also note that since counterbalancing of the conditions was implemented to minimize the temporal order effects in repeated measures design, the participants were randomly assigned to view either (**a**–**b**) the physical installation first, or (**c**–**d**) the VR reconstruction first.

The physical stimulus, the installation artwork, a spatial appropriation of the Mondrian’s room design by the artist Heimo Zobernig (heimozobernig.com), was exhibited in the Albertinum Museum in Dresden, Germany (skd.museum) as a part of the exhibition entitled Future Spaces: Kandinsky, Mondrian, Lissitzky and the abstract-constructivist avant-garde in Dresden 1919–1932. The digital stimulus, the VR reconstruction based on the same room design, was developed by our team using a modelling software called SketchUp (sketchup.com) and a game engine called Unity (unity.com). Both stimuli could be described as faithful interpretations of Mondrian’s room design from 1926 entitled Design Draft of Salon for Madame Bienert. Both versions had minor adjustments compared to the original design draft of Mondrian, as an artistic statement by Zobernig in the case of the installation (see Fig. 1a–b and Supplementary Fig. S2a), or as a reconstructive decision for the VR implementation to match it to the physical layout of the original room, the Damenzimmer in Ida Bienert’s villa in Dresden, for which it was designed (see Fig. 1c–d and Supplementary Fig. S2b). Briefly, Zobernig produced the artwork as an interpretation, which deliberately did not try to exactly reproduce Mondrian’s commissioned watercolour painting of the design, which furthermore did not match the actual, physical dimensions of the room in the Bienert Villa. Our team’s VR reconstruction was based on Mondrian’s composition combined with physical room measurements taken by us, and in line with Neoplasticist rules, slightly adjusting the design such as to fit into the actual room layout, including positions of walls, windows, and doors. In this sense, we did not aim to create identical architectural constructs, but compare the aesthetic experience in two very similar environments inspired by the same design idea. The adjustment of the VR design in accordance with the actual room dimensions led to VR dimensions of 499 by 494 cm, with a height of 360 cm, whereas the dimensions of the physical installation were 483 by 510 cm with a height of 385 cm, which followed Mondrian’s design sketch. Both the physical and VR installation incorporated monochromatic coloured patches for the room surfaces, and two main natural white lighting sources as an ambient light and as a surface light coming from the ceiling inside of the room, with no further controls for the similarity of the colour saturation and luminance. The outer environment surrounding the installation was a static grey scene in VR without any additional digital audio or digital avatars in the scene, whereas the physical installation was situated in the large museum space, right next to our VR installation (compare Fig. 1a–b and c–d). In both settings, some background noise from the visitors were inevitably present in the gallery space. Hardware and software used to record was a wearable eye-tracker (Tobii Pro Glasses 2) via Tobii Pro Glasses Controller, and a VR headset (HTC Vive with integrated Tobii eye tracker) via an executable file built by using Unity with Tobii Pro VR Analytics, a software package to enable data collection. In addition, an exit questionnaire was completed by participants, which included four items on basic demographic information, eighteen rating items as five-point Likert-scale that implement both positive and negative scoring on interest and opinions about art, and four open-ended questions as feedback (see Supplementary Fig. S1 for questions from the rating-scale questionnaire).

### Design

**Figure 2:**
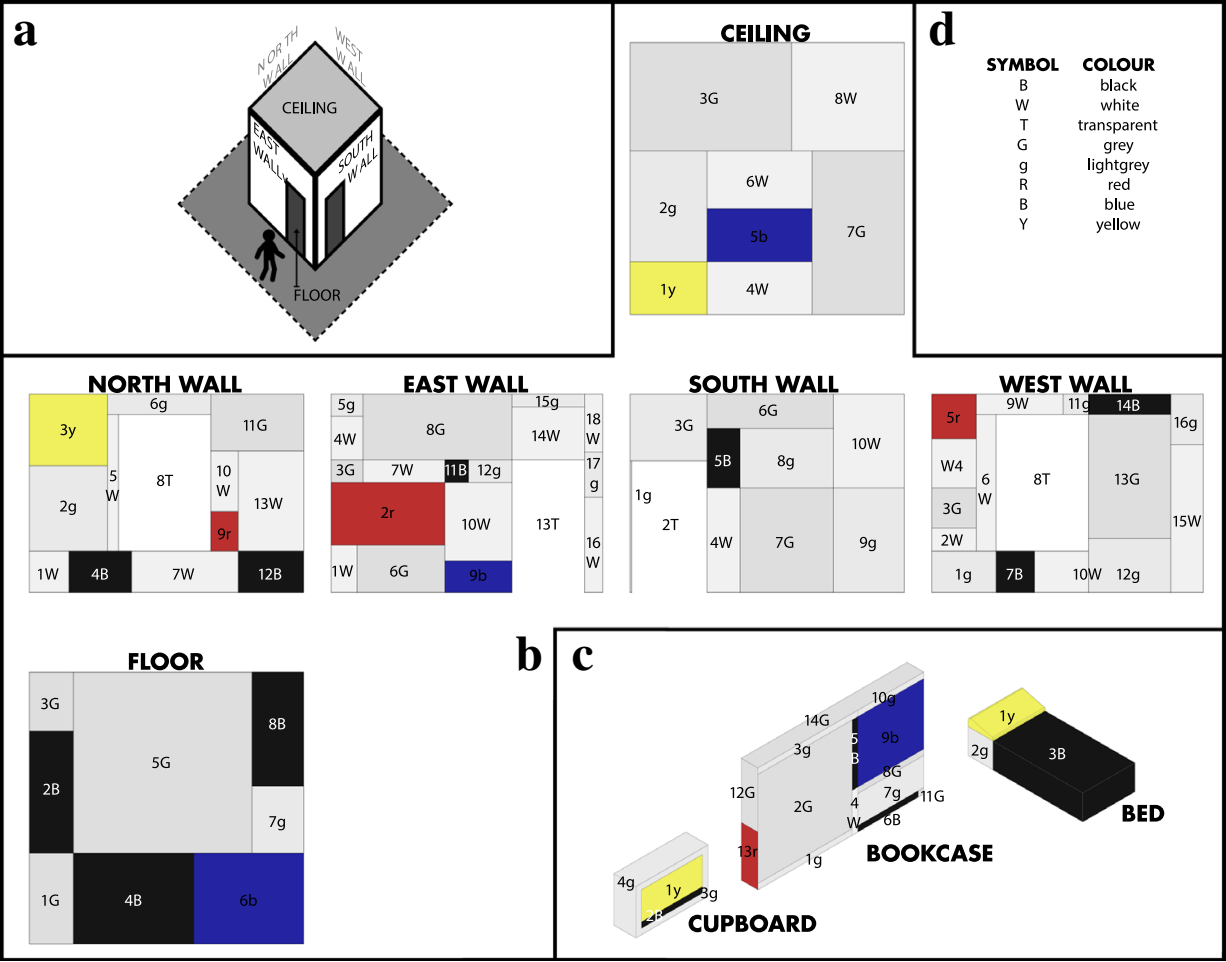
An overview of 3D AOI mapping for the VR condition. (**a**) A diagrammatic view of the cuboid room, in which (**b**) six surfaces of the room and (**c**) three pieces of furniture were present. Each individual coloured 2D panel was coded as an individual AOI during the development of the VR environment, and (**d**) had a unique colour value out of six possible colours. For example, the dwell time on red colour patches was calculated as the cumulative sum of fixation duration on four AOIs, namely North-9r, East-2r, West-5r, and Bookcase-13r. Along the same lines, the 3D AOI mapping was also formed for the physical condition, regarding the same sets of AOIs.

The study was designed as a within-subjects experiment, since each participant was expected to take part in both physical and VR conditions. To overcome carryover effects, the order of visiting the physical and virtual room was counterbalanced as the participants were pseudo-randomly assigned to either physical-first or VR-first conditions such that the half of the participants viewed the physical installation first, and the other half viewed the VR version first. The main conceptual justification for encountering two versions of the room design was the assumption that the forms of (top-down) effects due to the temporal order of viewing tend to cancel each out, albeit potentially introducing some noise to the data. Main dependent variables, both as absolute and area-normalised values, were dwell time defined as cumulative fixation duration, number of fixations, and average fixation duration on particular regions of the room, and all measured using the eye-trackers. Main independent variables were artwork media type as a binary variable labelled either as physical or virtual, and sets of AOIs as indexed by corresponding 3D geometry of the rooms, such as six surfaces of the cuboid room, three pieces of furniture, and six colours on room panels (see Fig. 2 for an overview of the AOI mapping). In line with the null hypothesis, no main difference is expected in terms of absolute and area-normalized dwell time, depending on the artwork media type and sets of AOIs. Note that although it was not explicitly recorded, we expected noise in the data from simply a re-exposure effect or forms of order effects such as fatigue, boredom, or practice, but aimed to minimize them using counter-balancing. Additionally, we assumed most participants engaged with the artworks for the first time during the experiment, at least for the first time during the data collection day, this assumption was supported by the fact that none of the participants mentioned about a previous viewing of the artwork, and a majority of them were not familiar with the artist as indexed by their response on the questionnaire.

### Procedure

After participants were given the written and oral instructions, they completed consent forms and screening forms about vision status at a reception desk, and they went through the experiment consisting of three steps. For one set of participants, they firstly were equipped with the mobile eye-tracking glasses and explored the physical artwork as long as they wanted by themselves. Researchers did not accompany participants, but since the study was conducted in a public museum space where other visitors had rights to view the artwork, there were a few instances of an additional visitor inside the installation, but only for brief intervals: there were five instances where another visitor was present simultaneously with a participant. For those five participants, the duration of co-presence was approximately 85 s in total, corresponding roughly to the 3% of the experiment’s whole eye-tracking recording duration inside the physical installation. Secondly, participants were equipped with the VR headset and explored the digital artwork, again without any time limitation. In this phase, a researcher always accompanied the participant simply to handle the cables between the headset and the computer. For the other set of participants, the order of viewing was reversed. For the physical installation, the participants always entered and exited the room using the same door opening on the South Wall. Due to the technical limitation of the physically walkable area in the VR version, participants started the VR experience at the centre of the VR room, and all were instructed to face forward towards the same wall at the start. Lastly, they completed a brief exit questionnaire either in German or in English depending on their preferred language at the reception desk (see Supplementary Fig. S3a for an overview of the procedure, and Supplementary Fig. S3b for a view from the gallery space). Note, eye-tracking calibrations were executed prior to the data collection, separately for the VR and mobile eye-tracker for each participant, to ensure the reliability of gaze data (in terms of precision and accuracy). The default in-built calibrations provided by the Tobii software (i.e., five-point for the VR and single-point for the mobile) were expected to provide a similar level of quality (but noisier than the data obtained in a lab setting from a research-grade screen-based eye tracker). No further interim recalibration, which is an occasionally used practice for eye-tracking drift correction for longer experimental designs, was carried out, because the installation was treated as a single stimulus, and the recording duration was short.

### Data analysis

Three sets of data were formed: from the questionnaire, mobile eye-tracking, and VR eye-tracking. All participants completed the physical part of the experiment; however, due to a data-saving error on the mobile eye-tracker data during the study, seven recordings could not be recovered, resulting in twenty-four valid recordings from eye-tracking glasses, instead of thirty-one. One participant did not participate in the VR part of the experiment due to discomfort caused by the screen brightness, resulting in thirty valid recordings from the VR headset. All participants completed the exit questionnaire, resulting in thirty-one respondents. Analysis of the questionnaire included descriptive statistics indicating the frequency distribution of rating responses on a Likert-scale.

The workflow from the raw recording data to the statistical analysis is explained in detail in Supplementary Fig. S4. The main difference in workflow between mobile and VR was the interim manual coding for the fixation locations, realized by the lead author (also see Supplementary Fig. S5 for a reliability comparison). Following on from that, the AOI mapping (as illustrated in Fig. 2) was the basis for five comparisons of eye-tracking data, related to the sparse spatial layout and composition style of Mondrian’s design, generating subsets of the data that were split out into separate levels for the data analysis: (1) Room elements consisted of three levels: interior cube surfaces, furniture, and regions representing the outside vista as door and window openings. (2) Colour types were split into two levels: luminance- and chrominance-type, such that whether the colour had luminance-information only as white, grey, black, or had chroma-information as red, blue, yellow. (3) Individual colours: all possible colours in the artwork produced six levels. (4) Cube surfaces contained six levels: ceiling, floor, along with north (N), east (E), south (S), and west (W) walls, where the directions of walls were approximately based on the original room location in Dresden. (5) Furniture comprised three levels: bed, cupboard, and bookcase. Note that minor topological differences between the physical and VR versions resulted in slightly different surface areas (see Supplementary Fig. S6 for visible surface areas corresponding to each set of AOIs).

Five main types of eye-tracking variables were analysed which were either reported as main results or summarized as supplementary results: (1) Absolute dwell time was defined as the cumulative fixation duration per AOI. (2) Area-normalized dwell time aimed to measure a type of fixation density or attentional density, after accounting for sampling at chance, to correct for the relative size of areas. It was calculated as cumulative fixation duration multiplied by the given AOI area in percentage, such that any given AOI was expressed as a fraction of the total area of all the AOIs. (3) Fixation count was the number of individual fixations on a given AOI. Unless there is a significant difference in the average fixation duration metric (see below), fixation count tends to be highly correlated with absolute dwell time. (4) Area-normalized fixation count was the normalized metric using area size of an AOI as a fraction, as above. (5) Average fixation duration was the mean duration of fixations on any given AOI, and a derivative metric since it was calculated as absolute dwell time divided by the number of fixations. This derived metric allowed comparison of how often the gaze is relocated between different image regions and viewing conditions (refer to Supplementary Fig. S7 for an overall table of mean and standard errors of the all above-mentioned measures, and also see Supplementary Fig. S8 for time to first fixation comparing the physical and VR settings for five comparisons, visualized as boxplots). Note that our explicitly presented results were mainly based on absolute and normalized dwell time to prevent analytical redundancy, since we expected very similar results for the potential analyses based on absolute and normalized fixation counts (see Supplementary Fig. S9 for the correlation table indicating the strength of the relation between dwell time and fixation count). Lastly, because free-viewing introduced a difference in viewing time between conditions and across participants, the viewing percentage can be calculated (see Supplementary Fig. S10).

Eye-tracking data for absolute and area-normalized dwell time were analysed using linear mixed-effects model (LME), which is equivalent to repeated measures analysis of variance (RM ANOVA). The reason not to run RM ANOVAs was that missing data from a single condition entail deletion of all data from the participant, whereas in LME each data point is treated as a single observation without participant exclusion. The main software packages used for the data analysis were MatLab, R, jamovi, Mathematica (mathworks.com, r-project.org, jamovi.org, wolfram.com); software for the data visualization were Unity, SketchUp, Lumion, Adobe CC (unity.com, sketchup.com, lumion.com, adobe.com).

Prior to evaluating our results, it is important to appreciate that we aimed to analyse a unique case study, mainly to compare the similarity of eye movement patterns between a physical and a virtual version of an art installation, and therefore our presented results and conclusions were limited within the confines of the real-world experimental conditions, rather than general and definitive. Therefore, the results could not be boldly generalized to the wider population and to wider forms of artworks; this limitation holds true for almost all research in empirical aesthetics. Additionally, as a general disclaimer, due to the small sample size in the traditional sense, this research has potentially low power, which in turn increases the probability of incorrectly failing to reject the null hypothesis and minimises the likelihood of reproducibility of results presented.

## Results

### Questionnaire

The eighteen Likert-scale rating questions can be clustered into four categories, and the most frequent response from five scale points as the mode of the data is often regarded as the most informative value (see Supplementary Fig. S11 for a summary of questionnaire responses). Overall, the questionnaire results indicated that the participants were highly educated, from diverse age groups, mostly regular museum visitors, were split into two on particular judgments comparing the two settings, open to VR experience, but only for shorter periods of viewing times. Note that the questionnaire was purely aimed at gaining more insight about participants’ views such as their overall attitudes towards visual arts and the experiment, or familiarity with the artwork. The main research question was about eye movements during the aesthetic experience, and not qualitative differences in the experience itself, and therefore particular assessment tools based on self-report (e.g., about art expertise^64^, aesthetic emotions^65^ or quality of VR use^66^) were not administered. On the conceptual level, we did not form our main hypothesis on the grounds of the confounding variables, for example, whether the amount of previous knowledge about the artist or an art period acts as a mediator on the relationship between dwell time and sets of AOIs (see Supplementary Fig. S12 for initial exploration of these relationships).

### Initial visualizations: dwell time as heatmaps

**Figure 3:**
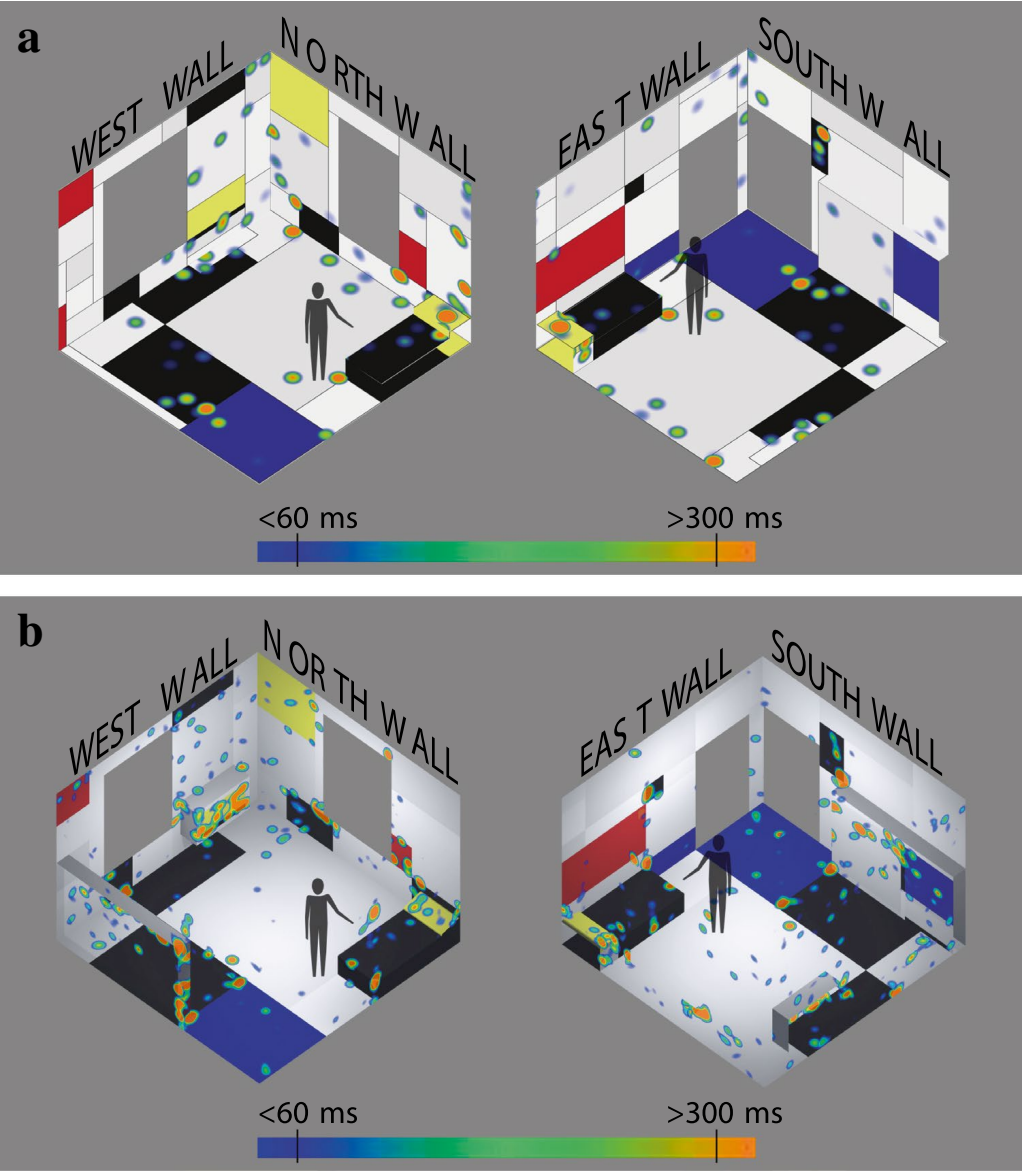
Exemplar fixation duration heatmap from a single participant. (**a**) Heatmap corresponding to the physical condition was formed after 89 s of interaction inside the physical installation, and (**b**) the heatmap corresponding to the VR condition was formed after 162 s of interaction inside the virtual environment. The colour-coded scale ranging from blue to orange approximately corresponds to fixation duration ranging from 60 to 300 ms.

Since both physical and VR versions were visited by the same participant group, an initial one-to-one qualitative comparison was possible, using heatmaps to visualise the amount of dwell time on any given point for physical and virtual environments. Note that both eye-trackers collected gaze data with approximately 100 Hz sampling rate and used the same algorithms to detect fixations. An exemplar dwell time heatmap pair from a single participant for both conditions and from two diagonal viewpoints of the room can be seen in Fig. 3. An initial qualitative evaluation indicated some similarities between overall viewing patterns and some specific differences such as response to furniture between physical and VR environments. Additionally, individual differences between participants were apparent: for example, some participants spent relatively more time in the artworks, resulting in longer total dwell times and fixation counts. Some variability was inevitably present in individual patterns of preference, for example, for particular colours or walls (see the open-data directory to view individual heatmaps of participants). Overall, in both conditions, hotspots of attention as indicated by densely fixated regions, seemed to be located on coloured patches of red, blue and yellow, as well as the furniture (see Supplementary Fig. S13 for gaze data validity measures).

### Total viewing time and fixations

**Figure 4:**
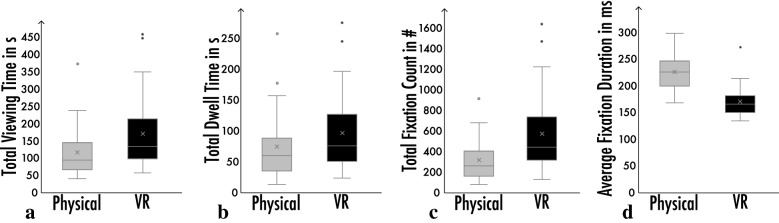
Main descriptive statistics comparing physical and VR environments: (**a**) total viewing time in seconds, (**b**) total dwell time in seconds, (**c**) total fixation count, and (**d**) average fixation duration in milliseconds illustrated as box-plots. Each box was drawn from first quartile (Q1) to third quartile (Q3) with a horizontal line denoting the median, and a cross denoting mean. Whiskers indicate minimum and maximum except outliers. Outliers were visualised as points ± 1.5 interquartile range. On average, participants spent about two minutes in physical installation and three minutes in virtual installation. Since viewing duration correlates with fixation duration and fixation count, the same trend of difference was observed both for dwell time and fixation count. However, the average fixation duration showed an opposite trend, where longer individual fixations were observed in physical installation compared to VR. The sample sizes were *N*_Physical_ = 24, *N*_VR_ = 30.

The statistical significance testing was carried out by using a general linear model. Main reported descriptive values were mean (M) and standard error of the mean (SEM) for any given analysis. The total time a participant spent in the room as indexed by the measured duration between entering into the installation and exiting from the installation was approximately two minutes in the physical environment and three minutes in the virtual environment (*M*_*Physical*_ = 118.50 ± 15.29 s, *M*_*VR*_ = 172.70 ± 19.51 s) on average, reaching a difference with statistical significance (*F*_(1, 52)_ = 4.43, *p* = 0.040, *η*_p_^2^ = 0.078), and shown in Fig. 4a. The viewing duration was comparatively longer than findings in the previous studies where the average viewing duration for 2D artworks such as paintings tends to be around 30 s in a museum context^67,68^, but rather similar to other research where the viewing durations for two distinct 3D installations were around two and four minutes^28^. In line with this total viewing duration difference, dwell time was relatively shorter in physical environment compared to virtual environment (*M*_*Physical*_ = 76.02 ± 11.59 s, *M*_*VR*_ = 97.68 ± 11.54 s), but did not reach statistical significance (as *F*_(1, 52)_ = 1.71, *p* = 0.197, *η*_p_^2^ = 0.032), and shown in Fig. 4b. Similarly, total fixation count was smaller in physical environment compared to virtual environment (*M*_*Physical*_ = 322.08 ± 42.32**,** *M*_*VR*_ = 579.13 ± 69.53), reaching statistical significance (as *F*_(1, 52)_ = 8.82, *p* = 0.005, *η*_p_^2^ = 0.145), and shown in Fig. 4c. Lastly, average fixation duration was substantially longer in physical environment compared to virtual environment (*M*_*Physical*_ = 226.25 ± 6.56 ms**,** *M*_*VR*_ = 171.31 ± 5.11 ms), reaching statistical significance (as *F*_(1, 52)_ = 45.05, *p* < 0.001, *η*_p_^2^ = 0.464), and shown in Fig. 4d.

Taken together, these initial data analyses showed that participants seemed to be slightly more engaged with the virtual installation compared to the physical installation, which might be attributed to several differences between the two conditions, including a possible novelty effect of VR as suggested by the questionnaire data showing that most participants had not used VR previously, and the presence of other visitors in the physical installation, among other possible distractions. The substantial difference between average fixation durations suggested a shift in terms of general viewing strategy: visitors seemed to be rapidly scanning the VR environment with shorter intervals of attentional focus as reflected by shorter fixations, compared to the physical environment.

### Spatial distribution of area-normalized dwell time

**Figure 5:**
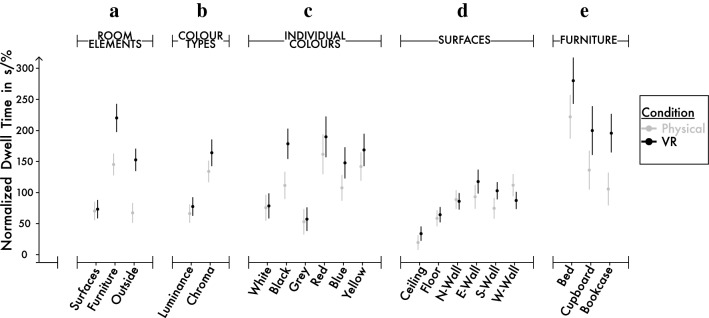
Graphs for normalized dwell time comparing physical and VR settings for five comparisons. (**a**) Room elements, (**b**) colour types, (**c**) individual colours, (**d**) surfaces, and (**e**) furniture. The x-axis shows the levels of AOIs, and the y-axis shows absolute dwell time in seconds. Physical and VR conditions were colour-coded as grey and black, respectively. The visualized data based on means, with whiskers indicating standard errors. The sample sizes were *N*_Physical_ = 24, *N*_VR_ = 30.

Each comparison of area-normalized dwell time (described as attentional density by accounting for sampling at chance to correct for the relative size of areas, and calculated as cumulative fixation duration multiplied by the given AOI area in percentage) was analysed using a separate linear mixed-effects model. For comparison 1 on room elements, all AOIs were used in the analysis, including AOIs belonging to outside areas visible through window and door openings. For comparison 2 and 3 on colour types and individual colours, every coloured surface was used in the analysis including AOIs on furniture and six faces of the cube, but not outside areas. For comparison 4 on cube surfaces, both furniture and outside were excluded from the analysis, as they were not part of the walls. For comparison 5 on furniture, only AOIs on three pieces of furniture was used in the analysis. Note that the cupboard as one of these furniture elements had four additional AOIs as its frame or profile in VR condition compared to the physical installation, since the cupboard was constructed as a 3D object in VR but rendered as only a 2D flat surface in the physical installation. Also note that each individual rectangular panel of the room was defined as a single AOI, and then they were combined into sets for a given analysis: for example, all four blue panels as four distinct AOIs in the room constituted blue-condition for the comparison 2 and 3 on colour types and individual colours, all panels on the ceiling constituted ceiling-condition for the comparison 4 on cube surfaces, etc. Post hoc comparisons using t-tests were Bonferroni corrected; significance level, denoted by *α*, was set to 0.05; and Bonferroni-corrected *p*-values as observed, unadjusted *p*-values multiplied by the number of comparisons made were reported for determining significance (*p*_corrected_ ⩻ *α*) for all results. Area-normalized dwell times comparing the physical and virtual environment without normalization of area covered by AOIs, are shown as boxplots in Fig. 5a–e. Lastly, note that the spatial distribution of absolute dwell time can be further seen as a supplementary analysis in Supplementary Fig. S14.

Comparison 1 on room elements: A significant difference between room elements was found, and a difference was observed between environments and in terms of an interaction: *F*_(2, 35.3)_ = 30.64, *p* < 0.001; *F*_(1, 30.1)_ = 7.14, *p* = 0.012; and *F*_(2, 80.2)_ = 10.34, *p* < 0.001 respectively. In terms of room elements, the area-normalized dwell time on furniture (*M*_*Furniture*_ = 185.70 ± 17.69 s/%) was higher than both for surfaces (*M*_*Surfaces*_ = 71.60 ± 7.46 s/%) and outside (*M*_*Outside*_ = 115.00 ± 13.09 s/%): *t*_(28.7)_ = 7.79, *p* < 0.001; and *t*_(28.6)_ = 5.11, *p* < 0.001, respectively. In terms of environments, the area-normalized dwell time in VR (*M*_*VR*_ = 148.64 ± 13.04 s/%) was longer than the physical installation (*M*_*Physical*_ = 93.50 ± 9.07 s/%). After normalizing for the area sizes, overall, the density of visual attention was highest in VR compared to in the physical environment, in line with the similar but non-significant trend observed for absolute dwell time. Here, the difference between VR and physical environment reached a statistical significance, mainly due to increased weighting of furniture and outside for the analysis, and also due to minor area size differences between physical and VR versions mentioned previously. Similarly, since the surface areas of furniture and outside were relatively smaller than room elements, the area-normalization changed the trend between room elements such that visitors attended significantly more densely on furniture of the installation compared to surfaces or outside, irrespective of environments. When the interaction was broken down by focusing on the two types of environment to check whether environmental differences exist for any level of room elements, normalized dwell time for furniture and outside were significantly higher in VR compared to the physical environment (*p* = 0.041, *p* = 0.011, respectively), whereas the difference was not present for surfaces (*p* > 0.05). When the interaction was broken down by focusing on the room elements to check how room elements differences have an effect differently for VR and physical environment, some trend changes were also visible, such as the normalized dwell time difference between surface_VR_ and outside_VR_ was significant (*p* < 0.001), but the dwell time difference between surface_VR_ and outside_Physical_ was not significant as (*p* > 0.05), suggesting that for some pairs, the amount of dwell time difference was dependent on the environment. Note that since the surface areas for both furniture and outside were relatively higher in VR condition, here, the area-normalization enhanced the dwell-time difference between environments and in terms of an interaction, whereas no significant difference was observed for absolute dwell time (compare Fig. 5a and Supplementary Fig. S14a).

Comparison 2 on colour types: A significant difference between colour types was found, but no difference was observed between environments or in terms of an interaction: *F*_(1, 35.1)_ = 42.701, *p* < 0.001; *F*_(1, 30.5)_ = 0.862, *p* > 0.05; and *F*_(1, 52.4)_ = 1.134, *p* > 0.05, respectively. The area-normalized dwell time on chroma-containing areas (*M*_*Chroma*_ = 153.00 ± 16.40 s/%) was higher than luminance-only areas (*M*_*Luminance*_ = 72.70 ± 7.02 s/%): *t*_(35.1)_ = 6.54, *p* < 0.001. Again, this shift of trends compared to absolute dwell time was a result of the relatively small area size of chroma-containing areas. Overall, in both environments, the density of visual attention was highest at red, blue, and yellow colours compared to black, grey and white.

Comparison 3 on individual colours: A significant difference between individual colours was found, but no difference was observed between environments or in terms of an interaction: *F*_(5, 53.4)_ = 9.840, *p* < 0.001; *F*_(1, 30.6)_ = 1.300, *p* > 0.05; and *F*_(5, 209.00)_ = 1.250, *p* > 0.05; respectively. After normalizing for the area sizes, overall, in both environments visitors attended most densely on red (*M*_*Red*_ = 184.69 ± 29.30 s/%), and least on grey (*M*_*Grey*_ = 55.60 ± 5.57 s/%).

Comparison 4 on cube surfaces: A significant difference between cube surfaces was found, no difference was observed between environments, and an effect was present in terms of an interaction: *F*_(5, 61.00)_ = 8.894, *p* < 0.001; *F*_(1, 30.8)_ = 0.232, *p* > 0.05; and *F*_(5, 234.2)_ = 2.648, *p* = 0.024, respectively. After normalizing for the area sizes, overall in both environments the density of visual attention was highest on east-wall (*M*_*East-Wall*_ = 112.00 ± 16.20 s/%), and lowest on the ceiling (*M*_*Ceiling*_ = 27.50 ± 4.26 s/%). The trend stayed the same compared to absolute dwell time, since the walls were roughly the same size within the cuboid rooms. When the interaction was broken down by focusing on the two types of environment to check whether environmental differences exist for any level of cube surfaces, all six post hoc comparisons yielded non-significant results (where all *p* > 0.05). The interaction was only pronounced, when the interaction was broken down by focusing on the cube surfaces to check how levels of cube surfaces have an effect differently for VR and physical environment: in this approach, some trend changes were visible, such as the normalized dwell time difference between ceiling_VR_ and south-wall_VR_ was significant (*p* < 0.001), but the normalized dwell time difference between ceiling_VR_ and south-wall_Physical_ was not significant (*p* > 0.05), suggesting that for some paired cube surfaces, the amount of dwell time difference was dependent on the environment.

Comparison 5 on furniture: A significant difference between types of furniture was found, but no difference was observed between environments or in terms of an interaction: *F*_(2, 31.09)_ = 6.28, *p* = 0.005; *F*_(1, 30.05)_ = 3.95, *p* > 0.05; and *F*_(2, 71.53)_ = 0.26, *p* > 0.05, respectively. In terms of furniture and after normalizing for the area sizes, the bed attracted highest attentional density *(M*_*Bed*_ = 252.61 ± 25.12 s/%) compared to the bookcase (*M*_*Bookcase*_ = 155.51 ± 17.75 s/%), and compared to the cupboard (*M*_*Cıpboard*_ = 175.02 ± 29.52 s/%): *t*_(28.29)_ = 3.43, *p* = 0.006; and *t*_(28.48)_ = 2.50, *p* = 0.043, respectively. Note that since the surface areas for furniture were slightly different between VR and physical conditions such as in VR condition the cupboard had four additional AOIs and therefore had more surface area, here, the area-normalization diminished the dwell-time difference between environments (*p* = 0.058), whereas a significant difference (*p* = 0.009) had been observed for absolute dwell time (compare Fig. 5e and Supplementary Fig. S14e).

## Discussion

The main research objective was to develop a methodology to assess the active exploration patterns of visual arts experience, and more specifically, to make a first step towards exploring the effects of an artwork’s presentation medium as physical or virtual on this experience. We have focused on one example artwork, and tested a limited number of participants, and indisputably, future work should draw on more targeted and possibly larger samples and a wider spectrum of artwork. Nevertheless, we are dealing with a large data set consisting of approximately 25,000 fixations, each of which represents a single, albeit small and relatively unconscious decision about the artwork. As it stands, our case study also aimed to demonstrate that empirical approaches can contribute in a meaningful way to the understanding of art appreciation and its delivery through different media. To the best of our knowledge, this is the first case of a direct and quantitative experiment to compare real-world aesthetic experience side-by-side with its VR counterpart. A major empirical justification of this research can be linked to communicating historic and contemporary visual arts to a remote audience^69–72^, especially in the context of novel trends in presenting arts to remote audience in the wake of the COVID-19 pandemic.

Our main conclusion following the overall results was that when engaging with a spatial art installation derived from the Mondrian’s design, participants showed predominantly similar viewing patterns on average in both physical and virtual environments, as indexed by gaze data from eye trackers. Our assessment on the similarity was the interpretation of the absolute and area-normalized dwell time analysis, which showed mostly non-significant main effects of environment and a lack of significant pairwise differences between the physical and VR versions for any significant interactions, except for absolute dwell time on some furniture elements (but also note that the furniture elements occupied only about ten percent of the surface area of the whole installation, and the most prominent design difference between physical and virtual installation was also present for furniture, in particular for the cupboard was a 3D piece in the virtual installation, but a 2D projection in the physical installation). In line with our expectations, our findings favour the null hypothesis, since no major difference was observed for the visual exploration patterns between in-situ and VR condition. It is important to briefly restate that, in general, the null results do not necessarily mean the lack of an effect or a difference, and they might be prone to over-interpretation; therefore, the findings can be described as preliminary evidence in need of further research and converging results.

Potential drivers for some particular gaze trends should be considered: (1) Irrespective of the viewing context, chroma-containing colours attracted higher visual attention densely compared to the luminance-only colours, as indexed by normalized dwell time. If the abstract nature of the installation and the minimum amount of semantic information available to the observer in this environment indicate that the participants’ visual attention was mostly driven by the bottom-up factors, then we can argue for that even an elementary saliency map based on colour or contrast should have a strong effect on the difference on attended locations (see Supplementary Fig. S15 for exemplar saliency maps generated using Itti algorithm^73^ and using histogram contrast). (2) The most prominent semantic information available was the types of furniture. This was only true, if a participant was able to attribute objecthood status to the rather atypical furniture elements in the room. In this sense, object-based attention as a higher-level cognitive process and often studied along with scene perception and semantically-driven saliency maps associated with it, can further help to explain some observed behaviours: for example, as a specific AOI within the set of furniture, the cupboard in the physical condition had the least amount of dwell time. Although it mainly consisted of yellow and black coloured patches, the cupboard was a flat 2D surface in the physical setting but not in the VR, which might reduce participants’ ability to recognize the flat surface as a piece of furniture, and therefore potentially diminishes the object-based attentional guidance. (3) In terms of six surfaces, although the ceiling attracted the least amount of attentional density as indexed by normalized dwell time, no statistically significant difference was observed between the four cardinal walls (N-E-S-W). This non-significant effect on the cardinal directions was also present in a similar, previous pilot study^29^, where we had utilised a mobile eye-tracker within another abstract installation consisting of coloured patches of parallelograms, covering all four walls of a gallery room. Additionally, a related observation from the present experiment, in both the physical and VR conditions, was that whilst participants were moving within the installation, the participants tended to not rotate themselves continuously, and did not form any number of full rotational circles in either clockwise or anti-clockwise directions. Put differently, the cumulative sum of a participant’s rotation on the axial plane parallel to the floor was almost always ± 180° in the physical installation since they entered and exited the installation from the same door; and very often within the range of ± 180° in the VR. We speculate that this observed behaviour of self-restriction on rotation might have an equalising effect on the distribution of visual scans on cardinal directions, and therefore on the normalized dwell time corresponding to the cardinal directions. Although the raw data recorded from simple gyroscopes in eye-trackers without precise motion tracking are not suitable for comparison, the general movement of participants, such as gait dynamics, might be prone to change depending on the exposure to the environment^74^. Here, as an anecdotal observation, participants naive to the VR tended to move more carefully or relatively slowly, compared to the physical world, a major factor might be the lack of visual bodily cues in the VR (also see Supplementary Fig. S16 for exemplar motion trajectories in VR).

In terms of experimental validity, most (if not all) empirical research in vision science has to make an inevitable trade-off between internal and external validity: internal validity roughly refers to the strength of the link between research findings and design of the study, and it can be increased for example by minimizing confounding variables and presenting well-controlled stimuli. On the other hand, external validity is related to the generalisability of the findings beyond the selected artificial stimulus, testing environment, or group of participants in the research. As a related concept, ecological validity often refers to the generalisability of the findings to real-world settings^75^. Here, we favoured ecological validity: although collecting gaze data using mobile and VR eye-trackers inside 1:1 scale physical and virtual versions of the artwork in a counter-balanced order from the same group of museum visitors aimed to preserve internal validity to some extent, our testing environment was far away from artificial laboratory conditions, where for example strict control of participant's viewing distance to a well-calibrated monitor accompanied with desktop-grade eye-tracker with higher sampling rate is often regarded as a procedural norm. On the other hand, art galleries and museums can be described as ecologically valid conditions where visitors’ behaviour can be measured^53^, and these physical conditions are not well tested so far for VR.

Given the overwhelmingly similar pattern of eye movements in the two different environments, our results would suggest that using VR would be described as a suitable proxy for the aesthetic experience in gallery and museum settings. Describing eye-movements as an indicator and one of the few directly measurable components of aesthetic experience during artwork viewing is a common assumption behind many previous studies: often, researchers utilize eye-tracking as a meaningful tool to compare conditions or participant groups to answer their research questions, for example, (1) whether figure paintings and landscape paintings induce dissociable gaze patterns^76^, (2) whether expert and non-expert participants in visual arts form different oculomotor measures^77^, or (3) whether the overlap of museum visitors’ viewing pathways on two paintings can be indexed and compared^78^. Additional measures can also be incorporated in studies and researchers can ask, for example, whether motion-capture alongside the eye-tracking during viewing a figurative sculpture by museum visitors who are trained dancers or non-dancers can be a feasible metric for aesthetic and kinesthetic experience^79^. Here, we used fixation maps and derived metrics such as dwell time per AOI as one potential way of comparing physical and VR museum contexts, and our main justification behind this is that the conceptualization of fixation maps^80^ allows us to quantify the similarity of eye movement traces^81^. Note that we fully acknowledge that aesthetic experience as a highly complex process cannot be reduced to eye-movements, but nevertheless maintain that eye-movement metrics can be an essential measure to compare the interaction of viewers with an artwork.

However, the assumption of the ecological validity of VR still needs more rigorous test cases to become a generalizable argument. We compared some of the basic measures that may be used to relate to aesthetic experience in terms of attentional engagement. Apart from mostly comparable results on absolute and area-normalized dwell times, visitors spent relatively more time in the virtual environment compared to the physical environment. More specifically, the main eye-tracking results showed that in both conditions, (1) participants visually explored in all directions as all surfaces of the installation except for the ceiling, (2) preferred coloured parts of the installation over the non-coloured parts as indexed by area-normalized dwell time, and (3) often revisited the same location as indexed by fixation counts on a given AOI. Results from the exit-questionnaire indicated overall positive feedback from participants, and provided a comparison between physical and virtual artworks, where participants were generally split equally into two towards favouring either physical- or virtual-versions on various evaluations. Since the perception and judgment of art are highly subjective, individual differences both in terms of gaze patterns and questionnaire responses were inevitably present. Overall, our findings suggest that in the test case presented here, the virtual presentation of the artwork did not radically change the observers’ visual exploration.

Recently, a comparative study between physical and virtual settings for an art gallery was investigated, with a focus on using EEG and ECG to classify emotion recognition and type of environment^52^: relevant to our results, participants’ self-assessment ratings on arousal and valence were part of their study, and almost no difference was found between physical and virtual contexts for eight art pieces, except for valence rating on a single art piece. In another study conducted to compare VR-museum and 2D computer monitor settings, no difference towards artworks’ perceived quality and artistic quality was found, although the aesthetic experience of paintings was described as more intense in VR^82^. Similarly, virtual environments can enhance memorability to some degree: for example, one study investigated active and passive view of spherical, 360° movie clips (such that whether the viewpoint of footage is dependent to head-orientation of the participant or not) involving Rubens and Nicolas paintings displayed via a head-mounted display (HMD), and their findings indicated that viewers’ impression on paintings were described as more powerful and realistic in the active viewing condition^83^. Another study compared the memory recall and recognition between 360° pictures displayed on HMD and on a tablet, and their results favoured the VR-display over tablet-display^84^. Apart from some enhancing effects of VR, the presentation medium of artwork seemed to induce minimal change on observers’ experience.

Looking further afield than virtual art galleries, researchers have compared different examples of VR environments with their corresponding contexts to validate the feasibility of using VR as an empirical research tool: for example, comparing user experience in physical and virtual buildings in terms of architectural research showed that user ratings were mostly not affected between the two conditions, although some difference was present in atmospheric ratings such as boredom, attractiveness, and invitingness^85^. In another study, the perceived spaciousness of a room in VR was investigated, replicating the main findings of its counterpart experiment in a physical room^86^. Similarly, comparing participants’ evaluations such as perceived pleasantness, interest, excitement, complexity, and satisfaction between physical and virtual interiors in terms of architectural and lighting design yielded no significant differences^87^. In a rather different research area, using measures of perceived presence, attitude towards a video game, memory recall and recognition of brand placement in a 2D, 3D, and VR version resulted in higher levels of presence in VR context, whereas attitude towards the video game and recognition of the brands was not changed^88^. Overall, the indication of VR as a valid context for behavioural research seems to be echoed by many researchers.

Eye-tracking and oculomotor data as a tool for aesthetic research, albeit useful, must be used with caution^89^. Correlation between preference and gaze data such as total dwell time and first fixation on one hand implied the feasibility of using eye-tracking metrics as an indicator of observers’ aesthetic judgment^90^, on the other hand, observers’ ability to acquire the gist of a painting rather impressively in sub-second duration regime^91^ might suggest a redundancy of gaze data, and the prediction potential of fixation parameters towards aesthetic value has been also challenged^37^. In our study, we described observers’ eye-tracking data both as a measure of visual interest and as a similarity measure of aesthetic experience, assuming similar visual input to the observer leads to similar aesthetic experience. Linking oculomotor responses to aesthetic judgment more directly might require additional sources of data such as continuous aesthetic ratings^92^ or eye movement recording synchronized with event-related potentials^93^.

Total viewing time is inherently linked to the fixation count and dwell time (i.e., total fixation duration), but not necessarily to the average fixation duration: whilst it is logical to think that the increase of the viewing time is often linearly translated into the increase of fixation count and dwell time; generally, no radical change is expected in terms of average fixation duration. Although there might be various factors , including the novelty of the VR experience, our finding of a significant shortening of average fixation duration in VR (*M*_*VR*_ = 171.31 ± 5.11 ms) compared to physical installation (*M*_*Physical*_ = 226.25 ± 6.56 ms) might indicate that VR introduces a change of attitude towards aesthetic appreciation, since the intention to positively appreciate a set of paintings results in a greater number of fixations and lower average fixation duration, compared to the intention to negatively evaluate ^94^. Alternatively, this difference found between the two conditions might be interpreted as an effect of authenticity: although in our case both conditions were reconstructions of the original artwork presented in two different media, the potential effects of originality (such as whether an artwork is original, copy, or fake) on observer rating and gaze behaviour have been noted previously^95^, therefore it may be possible that visitors might have presupposed the VR condition a less authentic version of the artwork. Similarly, a potential arousal effect induced by the novelty remarked by participants, might be a factor accounting for the observed difference, since outside aesthetics research, changes in arousal states are often linked to changes in various gaze metrics such as average fixation duration^96^, pupil size^97^ or saccadic velocity^98^. Additionally, compared to the mobile eye-tracking, the VR eye-tracking is, in theory, more robust to challenging conditions such as rapid head movements and change of environmental illumination: these might affect the fixation detection algorithms, and partially account for the average fixation duration differences.

Previous research also indicated that Mondrian’s abstract painting entailed a high amount of visual search as indexed by, for example, the number of saccades compared to other paintings^99^. If we were to denote dwell time on AOI sets as an indicator of visual search, then our results suggest that physical and VR condition also resulted in mostly similar visual search strategies during the visual exploration. Speculatively, particular differences between the two settings in general viewing such as average fixation duration (or albeit nonsignificant, total dwell time), might be linked to the current state of the VR. VR was perceived as a novel experience by the participants during the experiment, and this novelty might be linked to, for example, spending more time in the virtual installation. In time, the resemblance between physical and virtual galleries is only expected to increase, and with diminished novelty effects, more comparable general results might be expected in future studies.

Although promising results and valuable insights were acquired, comparing physical and virtual art spaces is still in its early stages, and our research was not aiming to provide fully comprehensive answers and explanations. Conducting a comparative experiment using two parallel, equally valid reconstructions models in a museum setting can be seen as a unique opportunity, but our findings on the similarity of gaze patterns for only one single, very specific example of an abstract art installation, with a particular population sample, does not justify bolder conclusions and generalizations about the validity of VR-context, especially without further behavioural measurements. First and foremost, most of our participants are regular art gallery and museum visitors, but many are not familiar with VR. Therefore, the extent and amount of some visitors’ mental state of surprise especially during VR condition, or their awareness of wearing the mobile eye-tracker or the VR headset, and the possible influence of these aspects on exploration patterns remains unclear. A training phase for both wearable devices in future experiments might reduce novelty effects and the remaining discrepancy between conditions to some extent. It is clear that there is an enormous potential for more comprehensive work, both in a variety of methods and in the scope of arts presented. For example, to increase the inter-stimulus consistency, a rigorous photogrammetric workflow consisting of 3D imaging laser scanner in conjunction with readings from colorimeter measurement can be utilized to be the base of the virtual counterpart of any given static installation, preferably followed by the colour calibration processes of a VR-HMD, which would also require additional psychophysical testing. The methodological workflow might also include comparing gaze patterns with body motions indexed by gyroscopes during the experiment^100^; or alternatively, a change of experimental design might allow for precise control for motion and viewing duration, at a cost of reduced freedom (see Supplementary Fig. S17). The concept of peripersonal space^101^ might also help to develop a more comprehensive theoretical perspective. In future research, it would be useful to compare a complete exhibition between physical and virtual environments, instead of comparing just a single artwork. For the physical condition, a complete exhibition as a set of selected artworks in a dedicated gallery space might be provided. For the virtual environment condition, a well-controlled exact digital replica of the physical exhibition might be created, and ideally, use of an untethered HMD with inside-out position tracking might allow visitors to walk within the virtual exhibition without any constraints or without relying on alternative ways of VR locomotion such as teleportation. Additionally, an augmented reality (AR) version of the same exhibition would allow a ternary comparison between physical, VR, and AR conditions. Interacting with artworks as stimuli might allow for asking more fine-tuned research questions, related to memorability^102,103^ or effects of haptic feedback and visual cues to depth information^104^. 3D saliency maps as extensions of 360° saliency maps^105^ might be investigated to describe the extent of bottom-up influence of the environment on gaze behaviour. In terms of further data analysis, investigation of temporal dynamics^106–108^ might provide more in-depth results, using tools such as temporal scan path analysis, or adapting methods from graph theory and related fields (see Supplementary Fig. S18 as an initial exploration of such directions). As we step inside the world of virtual museums and gallery spaces, current directions of VR in terms of artistic expression, digital heritage, and empirical research remains wide open. Despite the need for more comprehensive future studies, our research can be seen as an important and promising starting point for comparing aesthetic experience between virtual and physical environments.

## Supplementary Information

Supplementary Information 1.

## Data Availability

All anonymised data are accessible via Open Science Framework for anyone who would like to re-analyse the data or run any form of additional analyses: www.osf.io/bgtpy.

## References

[1] Fechner GT (1876). Vorschule der Aesthetik.

[2] von Helmholtz H (1863). Die Lehre von den Tonempfindungen als physiologische Grundlage für die Theorie der Musik.

[3] Buswell GT (1935). How People Look at Pictures: A Study of the Psychology and Perception in Art.

[4] Yarbus, A. L. *Eye Movements and Vision* (Springer, 1967).

[5] Noll AM, Hill M (1965). Computer-generated three-dimensional movies. Comput. Autom..

[6] Sutherland, I. E. A head-mounted three-dimensional display. In *American Federation of Information Processing Societies Conference Proceedings* 757–764 (1968).

[7] Clay V, König P, König SU (2019). Eye tracking in virtual reality. J. Eye Mov. Res..

[8] Locher P, Tinio PPL, Smith JK (2013). Empirical investigation of the elements of composition in paintings: a painting as stimulus. The Cambridge Handbook of the Psychology of Aesthetics and the Arts.

[9] Vessel EA, Rubin N (2010). Beauty and the beholder: Highly individual taste for abstract, but not real-world images. J. Vis..

[10] Gregory RL, Gregory RL, Heard PF, Harris JP, Rose D (1995). Black boxes of artful vision. The Artful Eye.

[11] Goldstein EB, Brockmole JR (2017). Sensation and Perception.

[12] Rauss K, Pourtois G (2013). What is bottom-up and what is top-down in predictive coding?. Front. Psychol..

[13] Machotka P (1995). Aesthetics: if not from below, whence?. Empir. Stud. Arts.

[14] Wagemans J (2011). Towards a new kind of experimental psycho-aesthetics? Reflections on the Parallellepipeda project. -Percept.

[15] Bullot NJ, Reber R (2013). The artful mind meets art history: toward a psycho-historical framework for the science of art appreciation. Behav. Brain Sci..

[16] Leder H, Nadal MT (2014). years of a model of aesthetic appreciation and aesthetic judgments: the aesthetic episode—developments and challenges in empirical aesthetics. Br. J. Psychol..

[17] Menninghaus W (2015). Towards a psychological construct of being moved. PLoS ONE.

[18] Nadal M, Chatterjee A (2019). Neuroaesthetics and art’s diversity and universality. Wiley Interdiscip. Rev. Cogn. Sci..

[19] Carrasco M (2011). Visual attention: the past 25 years. Vis. Res..

[20] Theeuwes J (2010). Top–down and bottom–up control of visual selection. Acta Psychol..

[21] Carroll N (2002). Aesthetic experience revisited. Br. J. Aesthet..

[22] Chatterjee A, Vartanian O (2014). Neuroaesthetics. Trends Cogn. Sci..

[23] Makin A (2017). The gap between aesthetic science and aesthetic experience. J. Conscious. Stud..

[24] Iseminger, G. Aesthetic experience. In *The Oxford Handbook of Aesthetics* Vol. 1 (ed. Levinson, J.) 99–116 (Oxford University Press, 2005).

[25] Nanay B (2010). Attention and perceptual content. Analysis.

[26] Nanay B (2015). Aesthetic attention. J. Conscious. Stud..

[27] Ferretti G, Marchi F (2020). Visual attention in pictorial perception. Synthese.

[28] Pelowski M (2018). Capturing aesthetic experiences with installation art: an empirical assessment of emotion, evaluations, and mobile eye tracking in Olafur Eliasson’s “Baroque, Baroque!”. Front. Psychol..

[29] Gulhan D, Zanker J (2019). Exploring artwork in situ: empirical aesthetics making use of mobile eye tracking. Perception.

[30] Zanker, J., Stevanov, J. & Holmes, T. Mobile eye tracking in the Royal Academy: analysing the interaction with abstract paintings. In *The 40th European Conference on Visual Perception *212 (2017).

[31] Stevanov J, Zanker J, Holmes T (2019). Mobile eye tracking in the Royal Academy of Arts: analysing scanpath sequences in Jackson Pollock’s paintings. Perception.

[32] Heidenreich SM, Turano KA (2011). Where does one look when viewing artwork in a museum?. Empir. Stud. Arts.

[33] Walker F, Bucker B, Anderson NC, Schreij D, Theeuwes J (2017). Looking at paintings in the Vincent Van Gogh Museum: eye movement patterns of children and adults. PLoS ONE.

[34] Klein C (2014). Describing art—an interdisciplinary approach to the effects of speaking on gaze movements during the beholding of paintings. PLoS ONE.

[35] Tymkiw M, Foulsham T (2019). Eye tracking, spatial biases, and normative spectatorship in museums. Leonardo.

[36] Guntarik O, Garcia JE, Howard SR, Dyer AG (2018). Traces: mobile eye tracking captures user sensory experience in an outdoor walking tour environment. Leonardo.

[37] Isham EA, Geng JJ (2013). Looking time predicts choice but not aesthetic value. PLoS ONE.

[38] Heilig, M. L. Sensorama simulator. United States patent U.S. Patent No. 3,050,870 (1962).

[39] Fisher, S. S., McGreevy, M., Humphries, J. & Robinett, W. Virtual environment display system. In *Proceedings of the 1986 Workshop on Interactive 3D Graphics* 77–87 (1987). 10.1145/319120.319127.

[40] Scarfe P, Glennerster A (2015). Using high-fidelity virtual reality to study perception in freely moving observers. J. Vis..

[41] Wilson CJ, Soranzo A (2015). The use of virtual reality in psychology: a case study in visual perception. Comput. Math. Methods Med..

[42] Rothacher Y, Nguyen A, Lenggenhager B, Kunz A, Brugger P (2018). Visual capture of gait during redirected walking. Sci. Rep..

[43] Steinicke, F. *Being Really Virtual* (Springer, 2016).

[44] Tricart C (2017). Virtual Reality Filmmaking: Techniques & Best Practices for VR Filmmakers.

[45] Pangilinan E, Lukas S, Mohan V (2019). Creating Augmented and Virtual Realities: Theory and Practice for Next-Generation Spatial Computing.

[46] Duchowski A (2002). 3-D eye movement analysis. Behav. Res. Methods Instrum. Comput..

[47] Duchowski A (2017). Eye Tracking Methodology: Theory and Practice.

[48] Madary M, Metzinger TK (2016). Real virtuality: a code of ethical conduct. recommendations for good scientific practice and the consumers of VR-technology. Front. Robot. AI.

[49] Miller MR, Herrera F, Jun H, Landay JA, Bailenson JN (2020). Personal identifiability of user tracking data during observation of 360-degree VR video. Sci. Rep..

[50] Brieber D, Leder H, Nadal M (2015). The experience of art in museums: an attempt to dissociate the role of physical context and genuineness. Empir. Stud. Arts.

[51] Bertamini M, Blakemore C (2019). Seeing a work of art indirectly: when a reproduction is better than an indirect view, and a mirror better than a live monitor. Front. Psychol..

[52] Marín-Morales J (2019). Real vs. immersive-virtual emotional experience: analysis of psycho-physiological patterns in a free exploration of an art museum. PLoS ONE.

[53] Carbon C-C (2020). Ecological art experience: How we can gain experimental control while preserving ecologically valid settings and contexts. Front. Psychol..

[54] Noll AM (1966). Human or machine: a subjective comparison of Piet Mondrian’s “composition with lines” (1917) and a computer-generated picture. Psychol. Rec..

[55] McManus IC, Cheema B, Stoker J (1993). The aesthetics of composition: a study of Mondrian. Empir. Stud. Arts.

[56] Plumhoff JE, Schirillo JA (2009). Mondrian, eye movements, and the oblique effect. Perception.

[57] Johnson MG, Muday JA, Schirillo JA (2010). When viewing variations in paintings by Mondrian, aesthetic preferences correlate with pupil size. Psychol. Aesthet. Creat. Arts.

[58] Swami V, Furnham A (2012). The effects of symmetry and personality on aesthetic preferences. Imag. Cogn. Personal..

[59] Troy NJ (1980). Mondrian’s designs for the Salon de Madame B…, à Dresden. Art Bull..

[60] Stevanov J, Zanker JM (2020). Exploring Mondrian compositions in three-dimensional space. Leonardo.

[61] Dalbajewa, B., Wagner, H., Biedermann, H., Dehmer, A. & Wagner M. *Visionary Spaces: Kandinsky, Mondrian, Lissitzky and the Abstract-Constructivist Avant-Garde in Dresden 1919-1932* (Sandstein Verlag, 2019).

[62] Geisler, W. S. & Cormack, L. K. Models of overt attention. In *The Oxford Handbook of Eye Movements* (eds Liversedge, S. P., Gilchrist, I. & Everling, S.) 439–454 (Oxford University Press, 2011). 10.1093/oxfordhb/9780199539789.013.0024.

[63] Kaplan RM, Saccuzzo DP (2017). Psychological Testing: Principles, Applications, & Issues.

[64] Specker E (2020). The Vienna art interest and art knowledge questionnaire (VAIAK): a unified and validated measure of art interest and art knowledge. Psychol. Aesthet. Creat. Arts.

[65] Schindler I (2017). Measuring aesthetic emotions: a review of the literature and a new assessment tool. PLoS ONE.

[66] Kourtesis P, Collina S, Doumas LAA, MacPherson SE (2019). Validation of the virtual reality neuroscience questionnaire: maximum duration of immersive virtual reality sessions without the presence of pertinent adverse symptomatology. Front. Hum. Neurosci..

[67] Carbon C-C (2017). Art perception in the museum: how we spend time and space in art exhibitions. -Percept.

[68] Smith LF, Smith JK, Tinio PPL (2017). Time spent viewing art and reading labels. Psychol. Aesthet. Creat. Arts.

[69] Hoang TN, Cox TN (2018). Alternating reality: an interweaving narrative of physical and virtual cultural exhibitions. Presence Teleoperators Virtual Environ..

[70] Parker E, Saker M (2020). Art museums and the incorporation of virtual reality: examining the impact of VR on spatial and social norms. Converg. Int. J. Res. New Media Technol..

[71] Puig A (2020). Lessons learned from supplementing archaeological museum exhibitions with virtual reality. Virtual Real..

[72] Checa D, Bustillo A (2020). Advantages and limits of virtual reality in learning processes: Briviesca in the fifteenth century. Virtual Real..

[73] Itti L, Koch C, Niebur E (1998). A model of saliency-based visual attention for rapid scene analysis. IEEE Trans. Pattern Anal. Mach. Intell..

[74] Burtan D (2021). The nature effect in motion: visual exposure to environmental scenes impacts cognitive load and human gait kinematics. R. Soc. Open Sci..

[75] Bourne V (2017). Starting Out in Methods and Statistics for Psychology: A Hands-on Guide to Doing Research.

[76] Massaro D (2012). When art moves the eyes: a behavioral and eye-tracking study. PLoS ONE.

[77] Francuz P, Zaniewski I, Augustynowicz P, Kopiś N, Jankowski T (2018). Eye movement correlates of expertise in visual arts. Front. Hum. Neurosci..

[78] Balbi, B., Protti, F. & Montanari, R. Driven by Caravaggio through his painting. In *COGNITIVE 2016: The 8th International Conference on Advanced Cognitive Technologies and Applications* 72–76 (2016).

[79] Wiseman B (2019). Embodied viewing and Degas’s little dancer aged fourteen: a multi-disciplinary experiment in eye-tracking and motion capture. Senses Soc..

[80] Wooding, D. S. Fixation maps: quantifying eye-movement traces. In *ETRA ’02: Proceedings of the Symposium on Eye Tracking Research & Applications* 31-36 (2002). 10.1145/507072.507078.

[81] Wooding DS (2002). Eye movements of large populations: II. Deriving regions of interest, coverage, and similarity using fixation maps. Behav. Res. Methods Instrum. Comput..

[82] Janković, D., Jevremović, V. & Carbon, C.-C. Visual art in the digital age: about the art experience in VR vs ordinary displayed museum contexts. In *The 7th Visual Science of Art Conference* (2019).

[83] Hine K, Tasaki H (2019). Active view and passive view in virtual reality have different impacts on memory and impression. Front. Psychol..

[84] Ventura S, Brivio E, Riva G, Baños RM (2019). Immersive versus non-immersive experience: exploring the feasibility of memory assessment through 360 degree technology. Front. Psychol..

[85] Kuliga SF, Thrash T, Dalton RC, Hölscher C (2015). Virtual reality as an empirical research tool: exploring user experience in a real building and a corresponding virtual model. Comput. Environ. Urban Syst..

[86] Meagher BR, Marsh KL (2015). Testing an ecological account of spaciousness in real and virtual environments. Environ. Behav..

[87] Chamilothori K, Wienold J, Andersen M (2019). Adequacy of immersive virtual reality for the perception of daylit spaces: comparison of real and virtual environments. LEUKOS.

[88] Roettl J, Terlutter R (2018). The same video game in 2D, 3D or virtual reality—how does technology impact game evaluation and brand placements?. PLoS ONE.

[89] Nayak BK, Karmakar S, Rebelo F, Soares MM (2019). Eye tracking based objective evaluation of visual aesthetics: a review. Advances in Ergonomics in Design.

[90] Holmes T, Zanker JM (2012). Using an oculomotor signature as an indicator of aesthetic preference. -Percept.

[91] Locher P, Krupinski EA, Mello-Thoms C, Nodine CF (2008). Visual interest in pictorial art during an aesthetic experience. Spat. Vis..

[92] Isik AI, Vessel EA (2019). Continuous ratings of movie watching reveal idiosyncratic dynamics of aesthetic enjoyment. PLoS ONE.

[93] Fudali-Czyż A, Francuz P, Augustynowicz P (2018). The effect of art expertise on eye fixation-related potentials during aesthetic judgment task in focal and ambient modes. Front. Psychol..

[94] Park H, Lee S, Lee M, Chang M-S, Kwak H-W (2016). Using eye movement data to infer human behavioral intentions. Comput. Hum. Behav..

[95] Locher P, Krupinski E, Schaefer A (2015). Art and authenticity: behavioral and eye-movement analyses. Psychol. Aesthet. Creat. Arts.

[96] Simola J, Le Fevre K, Torniainen J, Baccino T (2015). Affective processing in natural scene viewing: valence and arousal interactions in eye-fixation-related potentials. Neuroimage.

[97] Bradshaw J (1967). Pupil size as a measure of arousal during information processing. Nature.

[98] Di Stasi LL, Catena A, Cañas JJ, Macknik SL, Martinez-Conde S (2013). Saccadic velocity as an arousal index in naturalistic tasks. Neurosci. Biobehav. Rev..

[99] Sharma YS, Chakravarthy BK, Chakrabarti A, Prakash RV (2013). How people view abstract art: an eye movement study to assess information processing and viewing strategy. ICoRD’13.

[100] Bonnet CT, Davin T, Hoang J-Y, Baudry S (2019). Relations between eye movement, postural sway and cognitive involvement in unprecise and precise visual tasks. Neuroscience.

[101] Bufacchi RJ, Iannetti GD (2018). An action field theory of peripersonal space. Trends Cogn. Sci..

[102] Damiano C, Walther DB (2019). Distinct roles of eye movements during memory encoding and retrieval. Cognition.

[103] Krokos E, Plaisant C, Varshney A (2019). Virtual memory palaces: immersion aids recall. Virtual Real..

[104] Harris DJ, Buckingham G, Wilson MR, Vine SJ (2019). Virtually the same? How impaired sensory information in virtual reality may disrupt vision for action. Exp. Brain Res..

[105] John B, Raiturkar P, Le Meur O, Jain E (2019). A benchmark of four methods for generating 360 degree saliency maps from eye tracking data. Int. J. Semant. Comput..

[106] Wu DW-L, Anderson NC, Bischof WF, Kingstone A (2014). Temporal dynamics of eye movements are related to differences in scene complexity and clutter. J. Vis..

[107] Marlow CA (2015). Temporal structure of human gaze dynamics is invariant during free viewing. PLoS ONE.

[108] Brielmann AA, Vale L, Pelli DG (2017). Beauty at a glance: the feeling of beauty and the amplitude of pleasure are independent of stimulus duration. J. Vis..

