# Supplementary material for: Similarity of gaze patterns across physical and virtual versions of an installation artwork
Source: Sci Rep. 2021 Sep 23;11:18913. doi: 10.1038/s41598-021-91904-x (PMC8460659; doi:10.1038/s41598-021-91904-x)
Supplement: Supplementary file 1 — Supplementary Information 1. [file 41598_2021_91904_MOESM1_ESM.pdf]

Similarity of gaze patterns across physical and virtual versions of an installation artwork

Doga Gulhan*, Szonya Durant, Johannes M. Zanker
Department of Psychology, Royal Holloway, University of London, UK
*doga.gulhan@rhul.ac.uk

Supplementary Figures

SCREENING FORM

DEMOGRAPHICS

Participant ID	Age	Sex	Education (Highest degree)	Occupation

VISION STATUS

**Questions**	Yes	No
Are you wearing corrections glasses or contact lenses?	o	o
Are you near-sighted (corrected for distant objects) or far-sighted (for reading)?	o	o
In a gallery, would you wear glasses or contacts?	o	o
Do you know your prescription?	o	o
Are you depending on bi-focal or multi-focal contact lenses?	o	o
Are you wearing corrections for ASTIGMATISM?	o	o
Did you have corrective laser surgery in the last 12 to 24 months (LASIK)?	o	o
Are you colour blind?	o	o
Do you know about any other visual disorder?	o	o
Do you know about any other neurological conditions that effect vision?	o	o

PRIOR KNOWLEDGE IN ART

**Art expertise**	Not at all	Slightly	Moderately	Very	Extremely
To what extend are you interested in modern art?	o	o	o	o	o
To what extend are you interested in Bauhaus?	o	o	o	o	o
Are you familiar with works of Piet Mondrian?	o	o	o	o	o
Are you familiar with works of Heimo Zobernig?	o	o	o	o	o

ADDITIONAL INFO

Do you think that is there any points to take into consideration prior to experiment?

EXIT QUESTIONNAIRE

VIEWS IN ART

**Opinions on art**	Strongly disagree	Disagree	Neither disagree nor agree	Agree	Strongly agree
Viewing experience in installation was enjoyable.	o	o	o	o	o
Viewing experience in VR was boring.	o	o	o	o	o
The VR environment felt like a real room.	o	o	o	o	o
Art museums and galleries are losing their significance.	o	o	o	o	o
All public art objects/spaces should be digitized and available online.	o	o	o	o	o
The VR reconstruction was more exciting than the real installation.	o	o	o	o	o

**Questions on art exposure**	Never	Seldom	Sometimes	Often	Always
How often do you visit art museums, galleries, or events?	o	o	o	o	o
How often do you view art digitally?	o	o	o	o	o
How often do you use art-related sources (such as books, journals or websites)?	o	o	o	o	o
How often do you pursue an artistic activity or a hobby (e.g. painting, photography, workshops)?	o	o	o	o	o
How often do you play video games?	o	o	o	o	o
How often do you use VR?	o	o	o	o	o
How long would you comfortably view arts in a gallery or museum?	≤15 mins o	~30 mins o	~1 hour o	~2 hours o	≥4 hours o
How long would you comfortably view arts in a VR environment?	≤15 mins o	~30 mins o	~1 hour o	~2 hours o	≥4 hours o

FEEDBACK

What did you like about the experiment?

Was there anything you did not like?

Can you please provide 3 to 6 words which best describe your experience in the VR environment?

Do you have any additional comments (e.g., a couple of keywords)?

Supplementary Figure S1. The screening form and the exit questionnaire (English version), presented here in a compact, alternative layout compared to the layout used during the experiment.

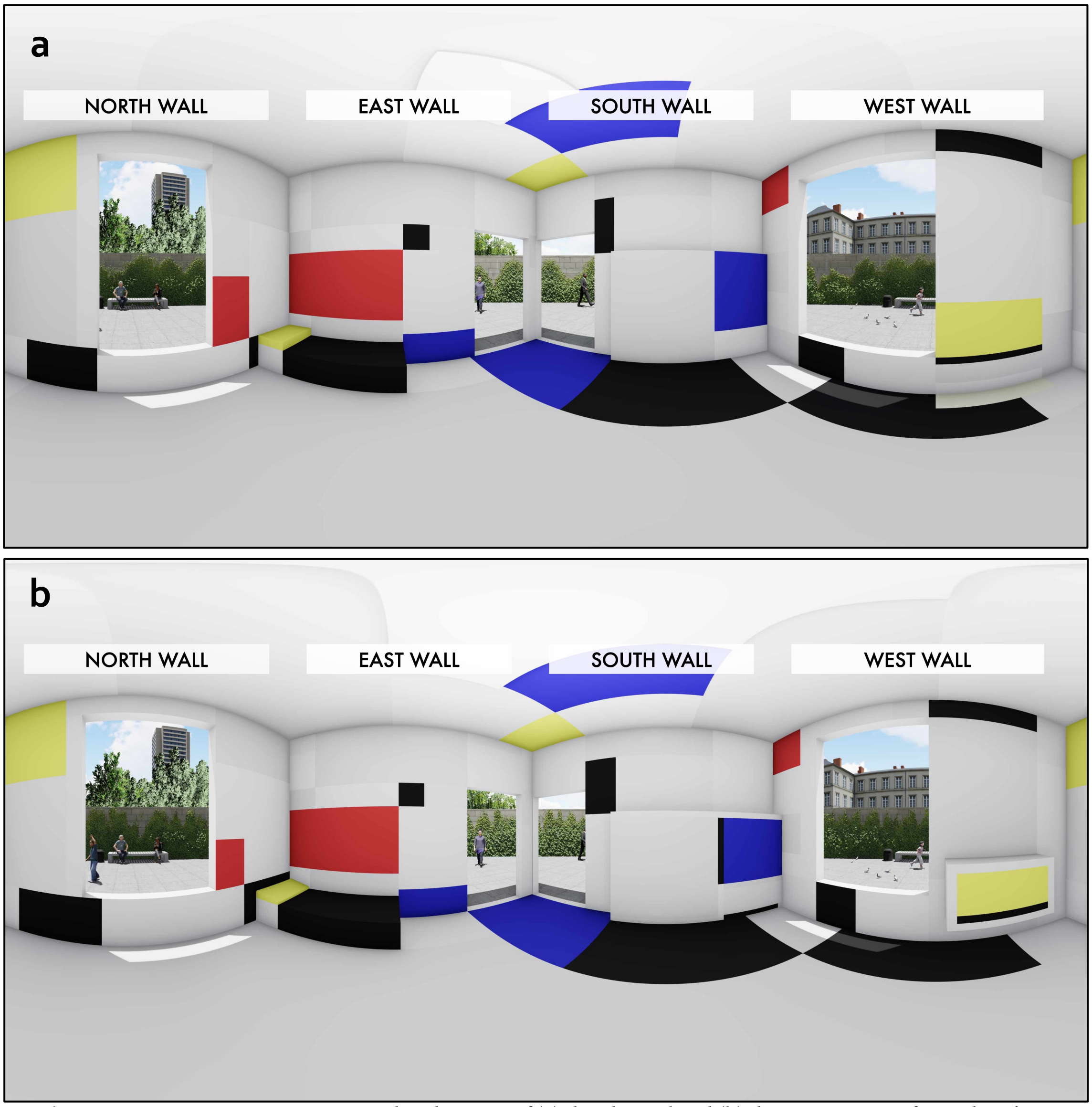

Supplementary Figure S2. Panoramic rendered images of (a) the physical and (b) the VR versions of Mondrian's room design. Note that, the exterior scene visible from the window and door openings are added to these renders to illustrate the scale of the installation. During the experiment, the visible exterior scene was the exhibition space of the Albertinum Museum in the physical installation, and a static grey scene in the VR.

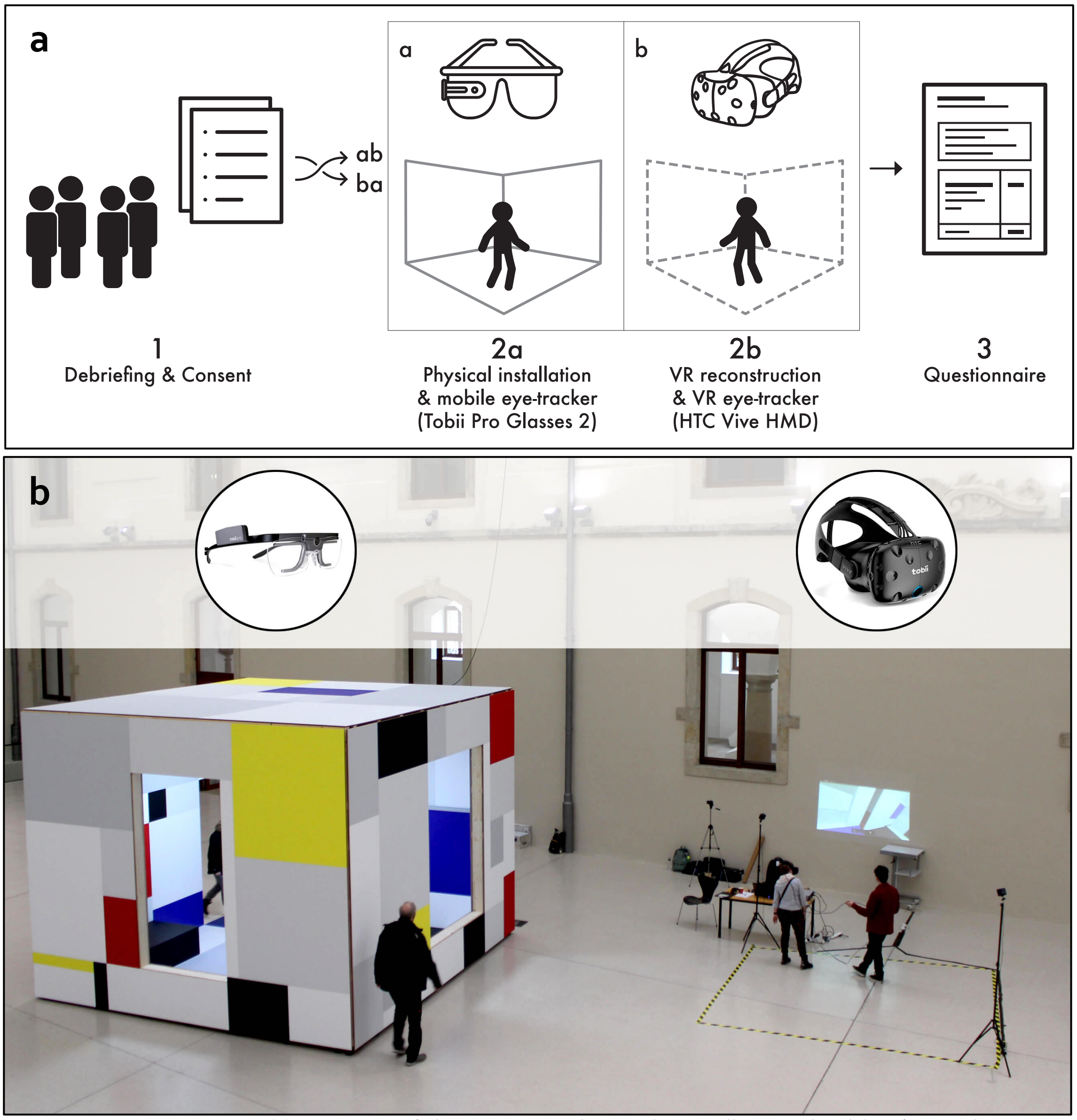

Supplementary Figure S3. (a) Overview of the experimental procedure. Following the debriefing and receiving consent, participants engaged with physical installation and digital reconstruction one by one, in a counter-balanced order. A wearable eye-tracker (Tobii Pro Glasses 2) wirelessly connected to a computer (Microsoft Surface) was used to collect data from the physical installation, whilst a VR eye-tracker (HTC Vive HMD with embedded eye-tracker) wired to another computer (Dell Alienware 15 R4) was used for the digital reconstruction. Note, the weight of the mobile eye-tracking glasses used for the physical installation was approximately 45 g, and the weight of the VR headset was approximately 470 g. Lastly, participants were asked to complete an exit questionnaire. (b) View from the gallery space. The physical installation can be seen on the left and the VR counterpart can be seen on the right, both situated side-by-side in the gallery. On top, the thumbnail images are showing the hardware used during the experiment: the mobile eye tracker (Tobii Pro Glasses 2) on the top-left, and the VR eye tracker (HTC Vive) on the top-right. Note that, the safe walkable area in the VR was limited to approximately 3.5 by 3.5 metres, regarding the optimum tracking area of the hardware. This inability of infinite walking in VR was mentioned to participants both before the experiment and during the experiment whenever needed, for example, when a participant has intended to exit from the doors in VR, or tried to get very close to the virtual walls.

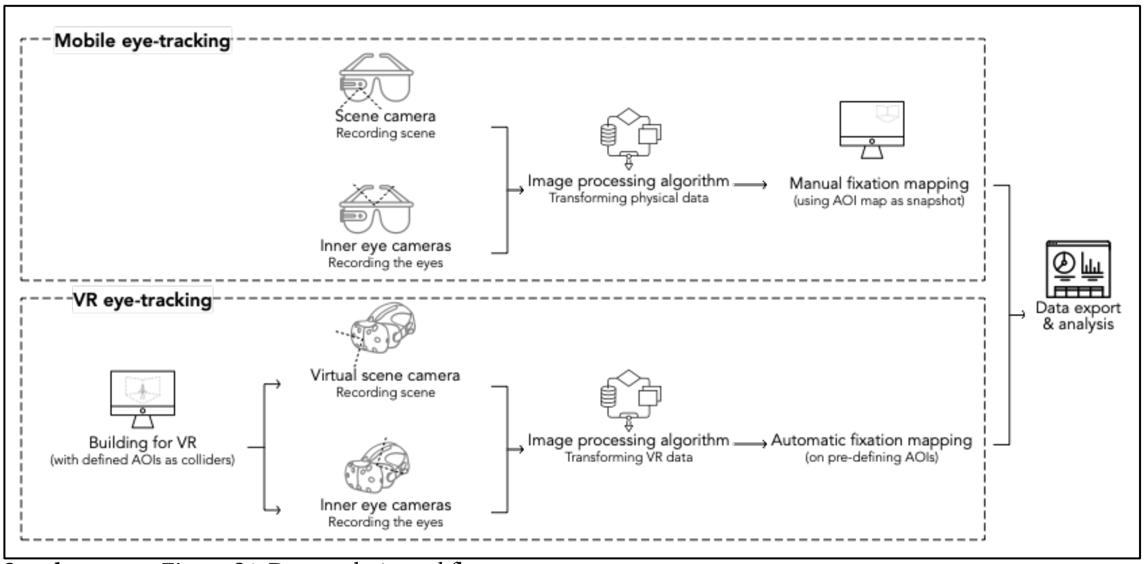

Supplementary Figure S4. Data analysis workflow.

For the mobile-eye tracking, the scene camera of the eye tracker captured complete video footage from the participant's point-of-view, whilst eye-tracking sensors with infra-red illuminators in the glasses recorded eye orientation, providing the direction of eye gaze. Tobii Pro Lab software matched the retinal coordinates of gaze to individual frames of the scene video. The velocity-threshold identification (I-VT) filter[1] was applied to classify gaze positions as fixations or other events, with a dispersion threshold of 1.0°, a minimum fixation duration of 60 ms, allowing for a gap of a maximum of 75 ms between fixations (namely, maintaining gaze on a single location is only defined as a fixation if the event lasted at least for 60 ms, and two or more consecutive fixations were combined into a single fixation if they were temporally separated by less than 75 ms), as suggested by[2], and by Tobii User's Manual for Glasses Pro 2). To localize the fixation positions, firstly we mapped manually the 3D interior of the installation to a 2D representation: this process can be defined as a backward texture mapping or an inverse UV mapping, since the 3D model's three-dimensional XYZ coordinates were projected back onto a 2D image as two-dimensional UV coordinates. Note that, U and V refer to the two axes of the 2D image; and X, Y, and Z refer to the three axes of the 3D model; and this notation simply aims to distinguish labelling of axes between 2D image and 3D model. As a single image file, this unwrapped version of the installation consisting of six faces of the installation stitched together was used as a reference image, which is often referred to as a snapshot in Tobii Pro Lab software. On top of the reference image, we added a second level of information called the AOI map (as illustrated in Fig. 2): the AOI map was a secondary image layer in which individual rectangular pieces of the installation were redrawn and tagged with unique identifiers. Each individually defined AOI had a set of features associated with it, such as colour and location, and whether it is a part of a specific wall, floor, ceiling, or furniture. Thus, we were able to manually code the fixation positions from the video footage to a single reference image. At this point, the real-world data output for each fixation had the duration, location, and corresponding AOI information. This interim manual coding was approximately 1/30X in real-time, e.g., for a 1-minute recording, the mapping took about half an hour. Being a time-intensive task, the coding was carried out by the lead author, but the reliability of coding (similar to the intercoder reliability) was initially checked with the additional independent coding by the last senior author for a single recording (see Supplementary Fig. S5 for comparison): given the overwhelming locational overlap between two mappings, this interim mapping was assumed to be minimally error-prone, and a complete set of secondary mapping was not sought after.

The workflow of eye-tracking data analysis from the VR eye tracker was similar, and comparatively simpler because there was already a direct allocation of gaze points to surface regions in the VR model, without a need for interim manual coding. During the design of the VR environment, individual surfaces in the 3D space of the digital installation were tagged in a similar way as when generating an AOI map for the physical setting. The oculomotor data were recorded by eye-tracking sensors with infra-red illuminators embedded within the VR headset, and the same fixation filter algorithm was applied, based on display screen coordinates in the headset. Localizing the fixation positions was an automatic process, since the positions of individual AOIs were already predefined (as illustrated in Fig. 2), and the fixation positions were converted to 3D XYZ coordinates inside the virtual environment. Therefore, the VR data output of fixations already contained the duration, location, and corresponding AOI information, without the need for an intermediary manual mapping procedure.

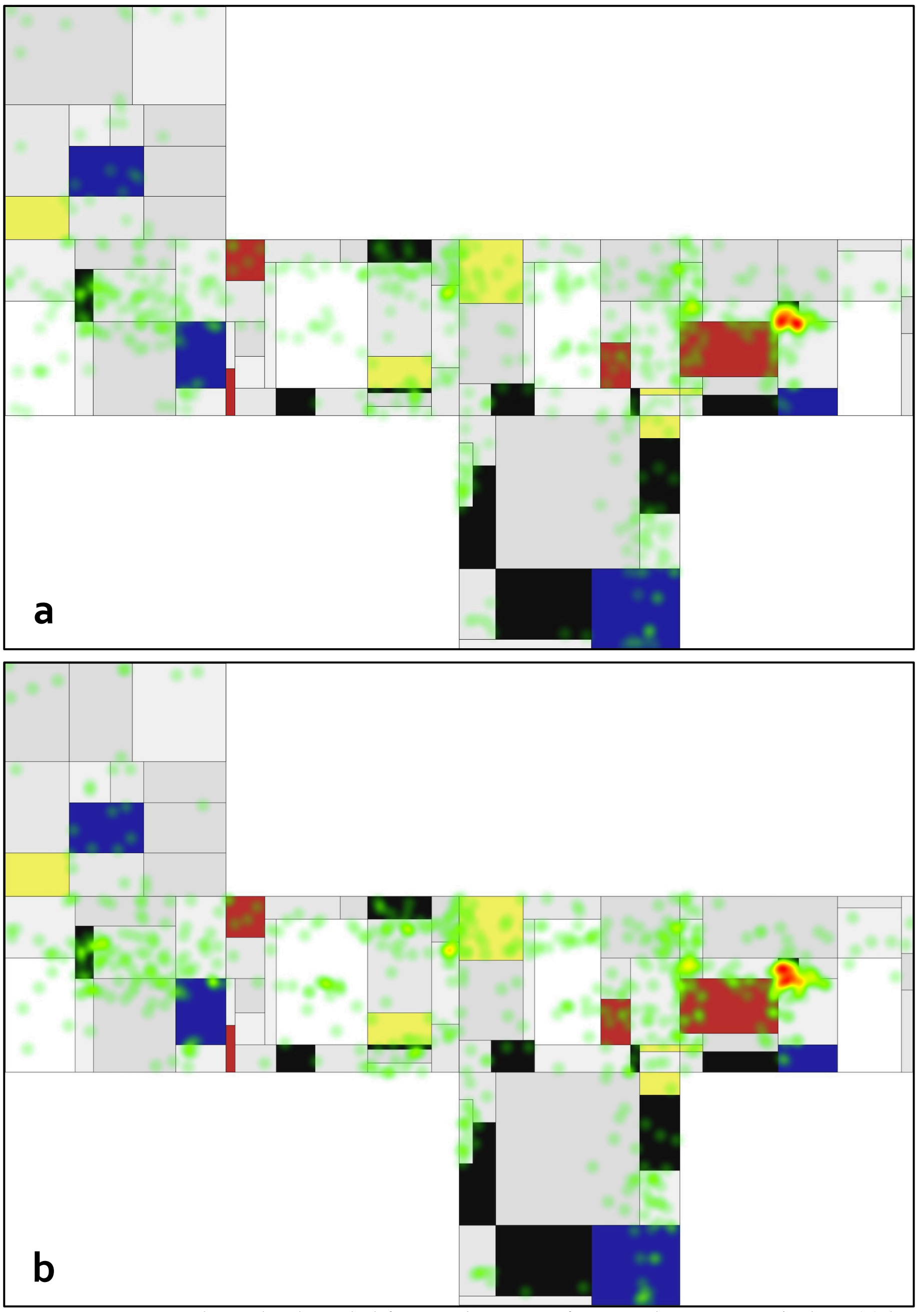

Supplementary Figure S5. Two independently coded fixation heatmaps for a single participant's data, (a) by the first author with a kernel sizes 25 pixels for the visualization and (b) by the last author with a kernel size of 30 pixels for the visualization, to visually inspect the reliability of the interim manual coding that was only required for the mobile eye-tracking data. Given the overwhelming locational overlap of localizing the fixations on particular AOIs, and given the time-consuming nature of this interim coding, all the remaining coding was carried out by the lead author without a secondary coder.

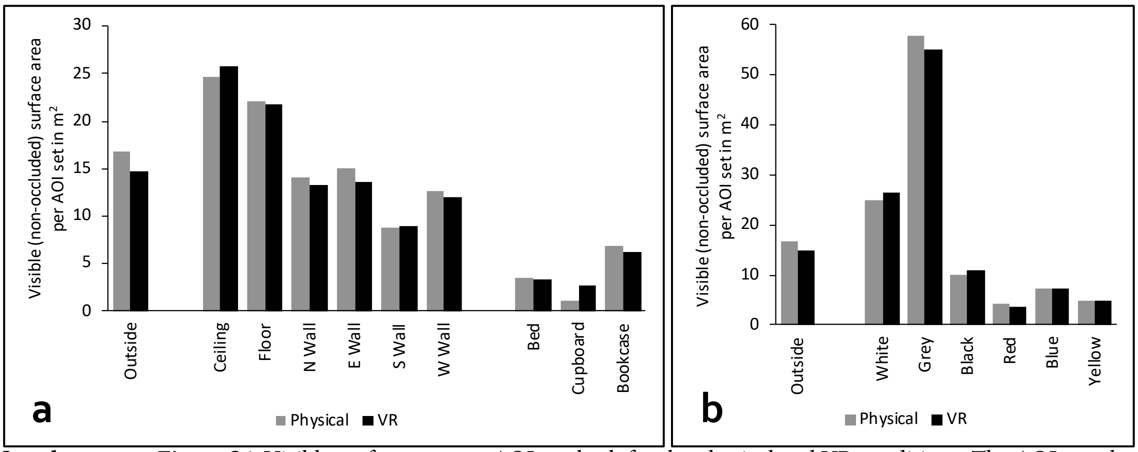

Supplementary Figure S6. Visible surface area per AOI set, both for the physical and VR conditions. The AOIs can be clustered together either (a) as a collection of outside (as window and door openings), six surfaces, and furniture elements; or (b) as a collection of outside and individual colours. In both instances, the total surface area of the physical installation was 125.727 m^2, and the VR counterpart was 122.648 m^2. Note that these minor discrepancies between two conditions have created a slight difference in terms of surface area, but the main analysis was based on area-normalized dwell time, which also aimed to normalize these minor discrepancies.

MEAN (± STANDARD ERROR OF MEAN)		AOI CONDITIONS																			
		ROOM ELEMENTS			COLOUR TYPES		INDIVIDUAL COLOURS						SURFACES						FURNITURE		
ENVIRONMENTS		Surfaces	Furniture	Outside	Luminance	Chroma	White	Grey	Black	Red	Blue	Yellow	Ceiling	Floor	N Wall	E Wall	S Wall	W Wall	Bed	Cupboard	Bookcase
PHYSICAL	**Dwell Time in s (Absolute)**	53.82 (±8.83)	13.09 (±1.66)	9.11 (±1.61)	49.00 (±8.05)	17.92 (±2.45)	15.45 (±2.90)	24.57 (±4.13)	8.98 (±1.34)	5.95 (±1.14)	6.19 (±0.72)	5.78 (±0.76)	3.82 (±0.63)	9.95 (±1.71)	10.37 (±1.67)	12.51 (±2.51)	5.50 (±1.26)	11.67 (±2.28)	6.01 (±0.75)	1.28 (±0.24)	5.80 (±0.86)
PHYSICAL	**Dwell Time in s/% (Area-normalized)**	69.48 (±11.40)	142.90 (±18.11)	68.10 (±12.03)	66.50 (±10.92)	138.39 (±18.91)	77.97 (±14.62)	53.51 (±9.00)	112.88 (±16.82)	178.09 (±34.01)	108.62 (±12.63)	147.83 (±19.39)	19.52 (±3.19)	56.48 (±9.69)	92.56 (±14.95)	104.25 (±20.87)	78.31 (±17.94)	116.38 (±22.74)	218.12 (±27.22)	143.84 (±26.59)	105.20 (±15.57)
PHYSICAL	**Fixation Count in # (Absolute)**	230.21 (±32.38)	51.33 (±5.72)	40.54 (±6.15)	209.67 (±29.58)	71.88 (±8.55)	66.92 (±10.28)	107.08 (±15.65)	35.67 (±4.89)	22.29 (±3.73)	27.00 (±2.63)	22.58 (±2.84)	20.29 (±3.00)	40.50 (±6.32)	44.54 (±6.24)	53.71 (±9.16)	23.25 (±4.57)	47.92 (±7.54)	21.50 (±2.19)	4.92 (±0.82)	24.92 (±3.31)
PHYSICAL	**Fixation Count in #/% (Area-normalized)**	297.17 (±41.80)	560.36 (±62.46)	303.18 (±45.99)	284.56 (±40.14)	555.19 (±66.01)	337.79 (±51.87)	233.22 (±34.09)	448.29 (±61.42)	667.30 (±111.68)	474.14 (±46.16)	577.45 (±72.75)	103.57 (±15.31)	229.77 (±35.85)	397.73 (±55.76)	447.41 (±76.31)	331.22 (±65.12)	477.94 (±75.22)	780.69 (±79.44)	551.93 (±92.38)	451.72 (±59.94)
PHYSICAL	**Fixation Duration in ms (Absolute)**	221.92 (±7.4)	247.79 (±8.7)	214.25 (±8.1)	221.83 (±7.2)	240.90 (±9.2)	213.99 (±7.0)	217.80 (±7.5)	247.26 (±12.7)	242.85 (±13.2)	221.43 (±9.6)	252.86 (±12.8)	183.51 (±11.0)	240.70 (±11.8)	222.80 (±8.5)	213.83 (±10.0)	222.25 (±10.7)	222.55 (±10.1)	274.42 (±13.1)	252.61 (±25.0)	222.53 (±9.2)
PHYSICAL	**Fixation Duration in ms/% (Area-normalized)**	286.47 (±9.53)	2704.95 (±95.19)	1602.23 (±60.40)	301.06 (±9.82)	1860.84 (±71.10)	1080.18 (±35.39)	474.34 (±16.23)	3107.79 (±159.59)	7269.69 (±394.55)	3888.58 (±167.99)	6465.54 (±326.39)	936.65 (±56.11)	1365.60 (±66.97)	1989.46 (±76.17)	1781.31 (±83.24)	3166.21 (±152.82)	2219.82 (±100.81)	9964.47 (±474.75)	28356.93 (±2802.76)	4034.29 (±167.27)
PHYSICAL	**Area in %**	77.47%	9.16%	13.37%	73.68%	12.95%	19.81%	45.92%	7.96%	3.34%	5.69%	3.91%	19.59%	17.63%	11.20%	12.00%	7.02%	10.03%	2.75%	0.89%	5.52%
VR	**Dwell Time in s (Absolute)**	57.06 (±7.80)	22.23 (±2.73)	18.39 (±2.29)	58.44 (±6.91)	20.85 (±3.22)	16.89 (±2.15)	25.70 (±3.19)	15.85 (±2.18)	5.65 (±1.36)	8.61 (±1.60)	6.59 (±1.21)	7.13 (±1.49)	11.45 (±1.99)	9.31 (±1.35)	13.07 (±2.69)	7.56 (±1.01)	8.55 (±1.22)	7.82 (±1.10)	4.43 (±1.08)	9.98 (±1.40)
VR	**Dwell Time in s/% (Area-normalized)**	73.30 (±10.02)	220.00 (±27.02)	152.60 (±19.05)	77.66 (±9.19)	164.23 (±25.39)	78.60 (±9.99)	57.25 (±7.10)	178.78 (±24.63)	189.96 (±45.68)	148.00 (±27.55)	168.84 (±31.01)	33.94 (±7.08)	64.39 (±11.18)	85.96 (±12.47)	117.66 (±24.25)	102.92 (±13.78)	87.32 (±12.48)	280.21 (±39.33)	199.97 (±48.68)	195.76 (±27.52)
VR	**Fixation Count in # (Absolute)**	348.90 (±47.52)	121.20 (±14.52)	109.03 (±12.74)	351.03 (±42.48)	119.07 (±18.34)	104.97 (±13.24)	158.13 (±20.11)	87.93 (±12.06)	33.37 (±7.45)	50.40 (±9.36)	35.30 (±6.10)	45.30 (±8.97)	67.23 (±12.05)	56.40 (±8.11)	82.73 (±15.74)	45.67 (±6.01)	51.57 (±7.07)	40.73 (±5.69)	23.77 (±5.25)	56.70 (±7.41)
VR	**Fixation Count in #/% (Area-normalized)**	448.19 (±61.04)	1199.44 (±143.67)	904.94 (±105.75)	466.45 (±56.44)	937.85 (±144.44)	488.35 (±61.59)	352.21 (±44.80)	992.04 (±136.06)	1122.43 (±250.58)	865.97 (±160.87)	904.46 (±156.28)	215.81 (±42.74)	378.11 (±67.78)	520.85 (±74.87)	744.68 (±141.64)	621.71 (±81.83)	526.70 (±72.18)	1459.89 (±203.77)	1071.64 (±236.89)	1112.46 (±145.31)
VR	**Fixation Duration in s (Absolute)**	165.34 (±4.10)	182.44 (±6.44)	169.46 (±7.08)	168.45 (±4.66)	174.67 (±5.79)	161.84 (±4.23)	163.60 (±4.93)	181.24 (±6.36)	163.95 (±5.36)	172.03 (±6.58)	179.66 (±7.91)	153.64 (±4.20)	166.37 (±5.64)	165.31 (±4.94)	153.42 (±5.71)	170.77 (±9.26)	164.63 (±5.56)	192.18 (±7.89)	171.18 (±8.91)	172.22 (±5.72)
VR	**Fixation Duration in s/% (Area-normalized)**	212.39 (±5.27)	1805.52 (±63.69)	1406.50 (±58.77)	223.84 (±6.20)	1375.83 (±45.60)	752.95 (±19.68)	364.38 (±10.99)	2044.67 (±71.70)	5515.19 (±180.46)	2955.86 (±113.11)	4449.77 (±248.73)	707.55 (±31.13)	935.63 (±31.74)	1475.72 (±67.32)	1380.89 (±51.37)	2324.91 (±126.11)	1681.53 (±56.79)	6887.60 (±282.72)	7204.16 (±517.73)	3379.01 (±112.25)
VR	**Area in %**	77.85%	10.10%	12.05%	75.26%	12.70%	21.49%	44.90%	8.86%	2.97%	5.82%	3.90%	20.99%	17.78%	10.83%	11.11%	7.35%	9.79%	2.79%	2.22%	5.10%

Supplementary Figure S7. Summary data table of eye-tracking metrics. The sample sizes were $N_{Physical} = 24$, $N_{VR} = 30$.

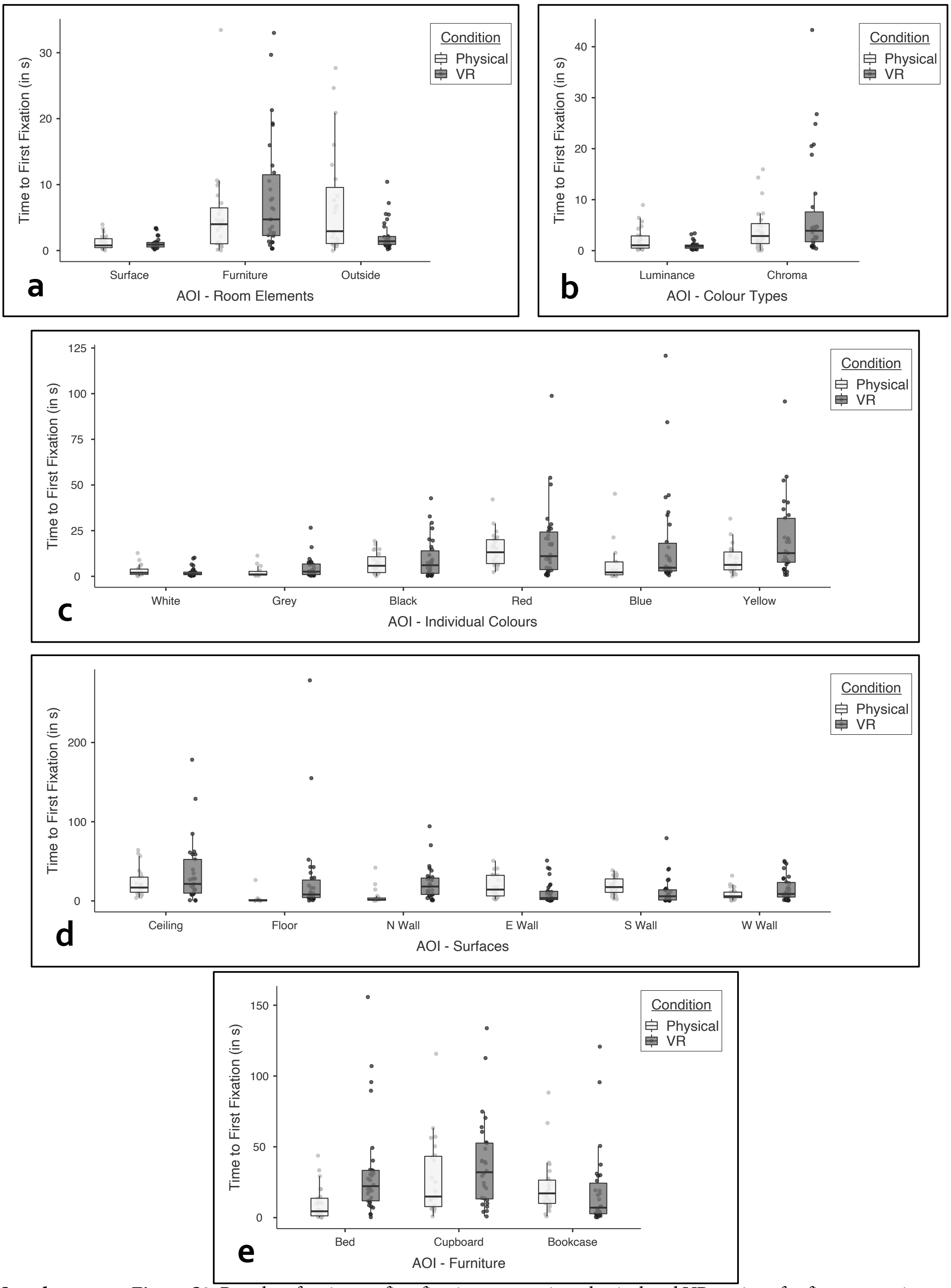

Supplementary Figure S8. Boxplots for time to first fixation comparing physical and VR settings for five comparisons: (a) room elements, (b) colour types, (c) individual colours, (d) surfaces, and (e) furniture. The x-axis shows the levels of AOIs, and the y-axis shows time to first fixation in seconds. Physical and VR conditions are shown as light and dark grey, respectively. The sample sizes were $N_{\text{Physical}} = 24$, $N_{\text{VR}} = 30$.

CORRELATION BETWEEN FIXATION DURATION AND FIXATION COUNT								
			CORRELATION COEFFICIENTS (Physical Condition, N = 24)			CORRELATION COEFFICIENTS (VR Condition, N = 30)		
			Pearson's r_P	Spearman's r_S	Kendall's τ	Pearson's r_P	Spearman's r_S	Kendall's τ
AOIs	Surfaces	Ceiling	.961***	.923***	.830***	.994***	.982***	.917***
		Floor	.982***	.955***	.876***	.990***	.989***	.927***
		N Wall	.982***	.958***	.859***	.978***	.987***	.923***
		E Wall	.986***	.959***	.854***	.989***	.942***	.846***
		S Wall	.984***	.976***	.896***	.970***	.914***	.788***
		W Wall	.976***	.975***	.889***	.986***	.972***	.904***
	Furniture	Bed	.897***	.929***	.796***	.956***	.962***	.852***
		Cupboard	.960***	.934***	.832***	.982***	.980***	.908***
		Bookcase	.965***	.951***	.847***	.970***	.974***	.892***
	Colours	White	.985***	.990***	.940***	.986***	.956***	.857***
		Grey	.987***	.953***	.860***	.988***	.966***	.869***
		Black	.945***	.932***	.788***	.980***	.966***	.871***
		Red	.985***	.986***	.934***	.991***	.977***	.900***
		Blue	.942***	.971***	.874***	.988***	.934***	.833***
		Yellow	.948***	.837***	.684***	.974***	.982***	.914***

Supplementary Figure S9. Correlation table indicating the strength of the relation between dwell time (as total fixation duration) and fixation count for sets of AOIs, summarized separately for physical and VR conditions. Both dwell time and fixation count can be treated either as continuous variables (and analysed using Pearson's r_P) or ordinal variables (and analysed using Spearman's r_S or Kendall's τ). Irrespective of the statistical standpoint, the data showed significant, positive, linear relationship for every AOI set: the numbers on the table denote the self-correlation coefficients (such as the correlation between dwell time on the ceiling and fixation count on the ceiling, etc.) corresponding to three different statistical measures, and *** denotes $p < .001$. Note that, since the area-normalized metrics were directly derived from the regular metrics, the dwell time and area-normalized dwell time has a perfect linear positive relationship with a correlation coefficient of +1. Similarly, the fixation count and area-normalized fixation count has also a perfect linear positive relationship with a correlation coefficient of +1. Hence, the correlation coefficients between area-normalized dwell time and area-normalized fixation count are the same as this table. One interpretation of these highly strong correlations is that the results of a potential set of supplementary analysis using absolute and area-normalized fixation count should be very similar to our main results in this study where we have chosen to use absolute and area-normalized dwell time. The sample sizes were $N_{Physical} = 24$, $N_{VR} = 30$.

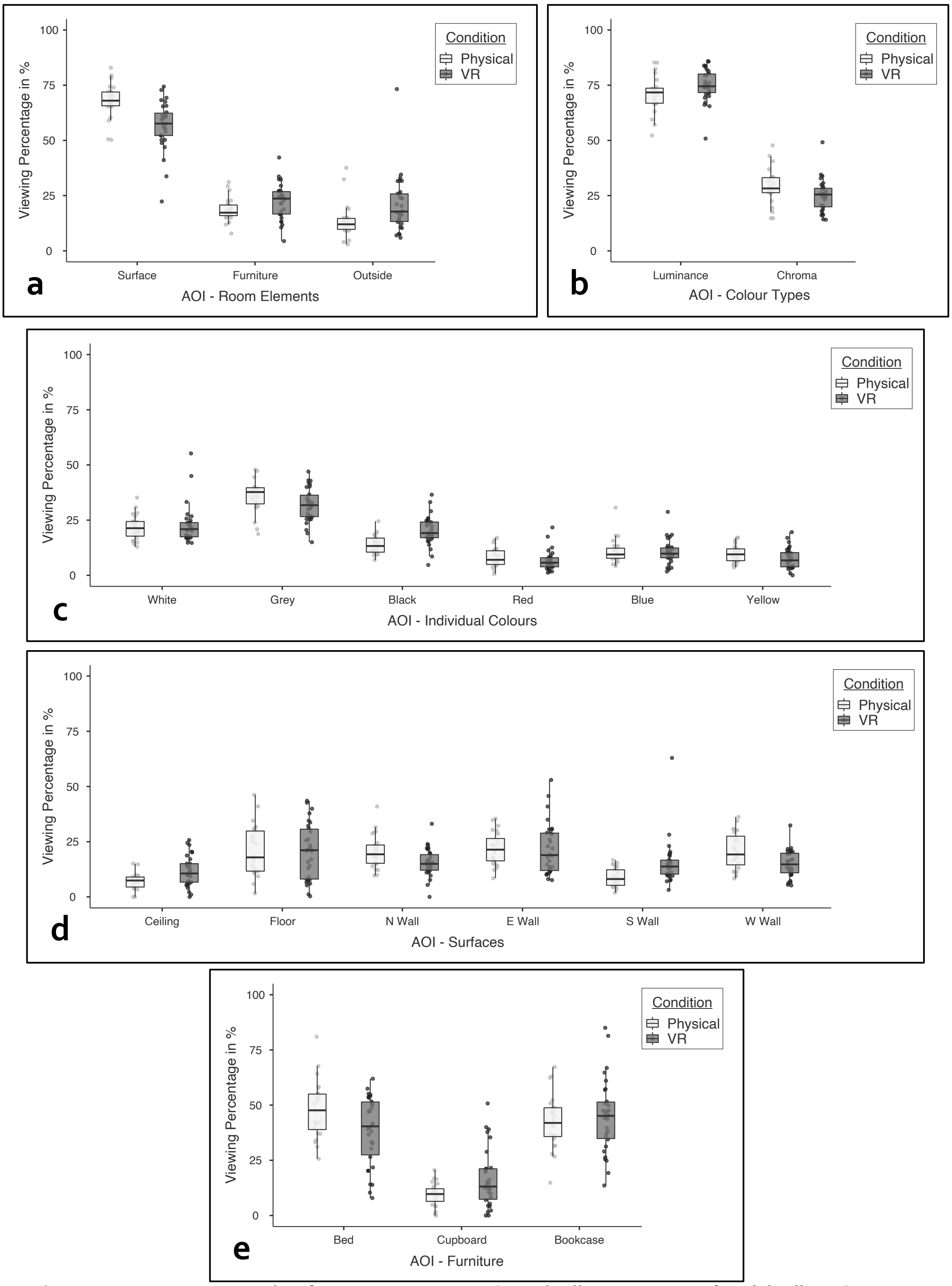

Supplementary Figure S10. Boxplots for viewing percentage (as % dwell time on AOIs of total dwell time), comparing physical and VR settings for five comparisons: (a) room elements, (b) colour types, (c) individual colours, (d) surfaces, and (e) furniture. The x-axis shows the levels of AOIs of a given grouping, and the y-axis shows the viewing proportions in %. Physical and VR conditions are shown as light and dark grey, respectively. The sample sizes were $N_{Physical}$ = 24, N_{VR} = 30. Note, particularly to compare the physical and the VR condition, the normalized data show an overlapping trend of viewing percentage between the two conditions, and is similar to the results of both the absolute and area-normalized dwell time analysis in this sense (see Fig. 5 and Supplementary Fig. S14, respectively).

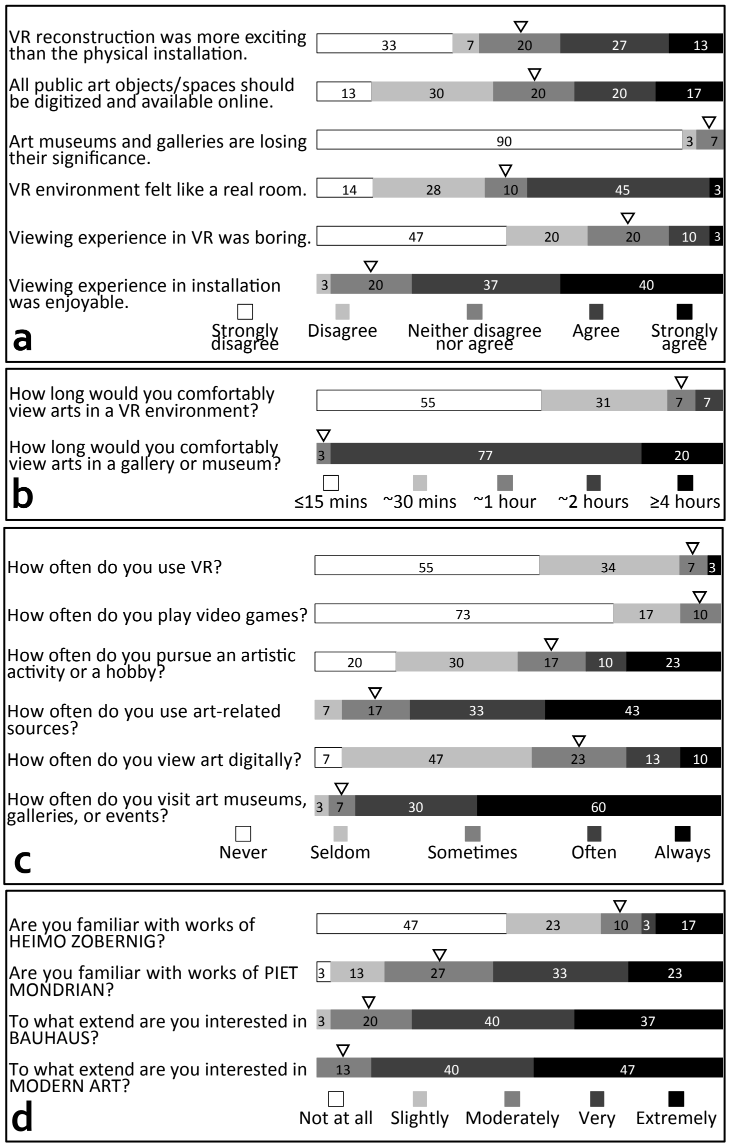

Supplementary Figure S11. Questionnaire results visualized as frequency plots, where numbers on the bars indicate the percentage of responses rounded to the nearest one, and the triangle above the bars indicates the mid-point of the middle response: (a) attitudes as agreement ratings, (b) comfort as ratings on preferred view duration, (c) exposure to art forms as frequency ratings, and (d) knowledge as familiarity ratings. The sample sizes were $N_{\mathrm{Physical}} = 24$, $N_{\mathrm{VR}} = 30$.

(a) Attitude ratings showed that visitors were split into two (40%:40%) on judging whether the VR reconstruction was more exciting than the physical installation. Similarly, the VR environment felt like a real room for around half of the participants (48%). A similar split was also visible on whether all public art should be digitized and available online (37%) or not (43%). At the same time, visitors strongly disagreed with the idea that the art museums and galleries are losing their significance (90%). The physical installation was enjoyable for most of the participants (77%), and the VR experience was not boring for most participants (67%). (b) Preferred viewing duration that participants would think to allocate to viewing artworks was radically different between two conditions: participants seemed to be comfortable to spend much more time in a gallery (77% for circa 2 hours) in comparison to VR (55% for less than 15 minutes). (c) Frequency responses on their exposure to art forms revealed that most of the participants were not VR users (55%) and did not play video games at all (73%). Visitors showed diverse responses on their pursuit of artistic activities and use of art-related sources. Around half of the visitors reported that they view art digitally either seldom or never (54%), whereas they were keen on visiting art museums, galleries and events very frequently (60%). (d) Level of interest in art periods and familiarity with artists was overall high: visitors were extremely interested in modern art (47%), and very interested in Bauhaus (40%). Visitors were very familiar with works of Mondrian (33%), but not at all familiar with works of Heimo Zobernig (47%).

Open-ended feedback revealed an overall satisfaction from the experience, and visitors described it using keywords such as novel, surprising, stimulating, realistic, exciting, interesting, impressed, curious, enriching, calm, etc. According to visitors, a prominent drawback of the study was the technical properties of devices, some participants particularly remarked on the spatial resolution of VR screen, limited field of view, and discomfort due to screen. In regards to the additional question about formal educational level (not presented here), the participants were biased towards higher education, as qualifications showed a highly skewed distribution among participants compared to the population: PhD (23%), graduate 2 (33%), graduate 1 (17%), secondary 2 (23%), and secondary 1 (3%).

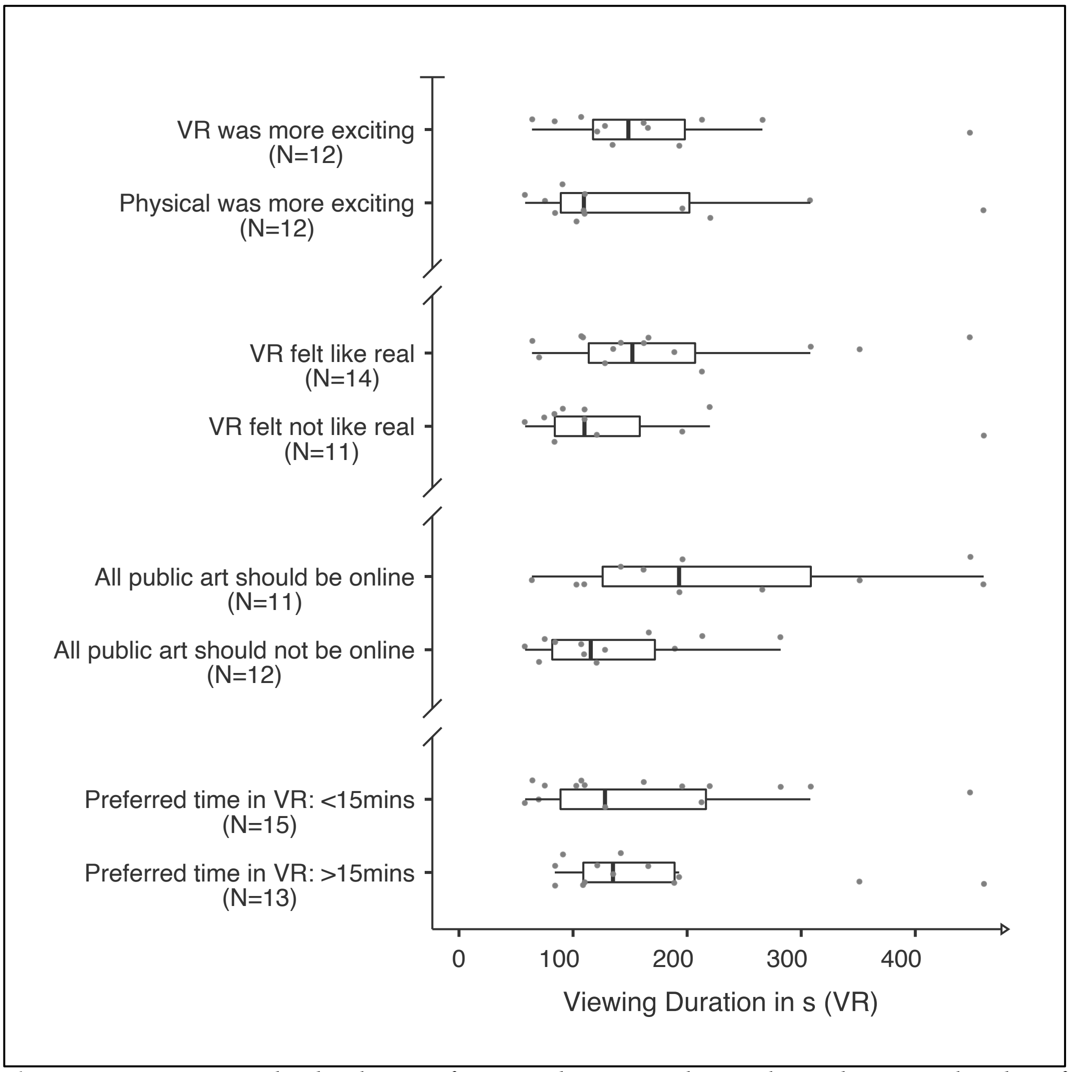

Supplementary Figure S12. The distribution of viewing duration in the VR, bisected into two based on four questionnaire item responses which resulted in close to bimodal splits. The x-axis shows the viewing duration in s for the VR condition, the categorically separated y-axis shows the response pairs of four items, with individual datapoints corresponding to individual participants.

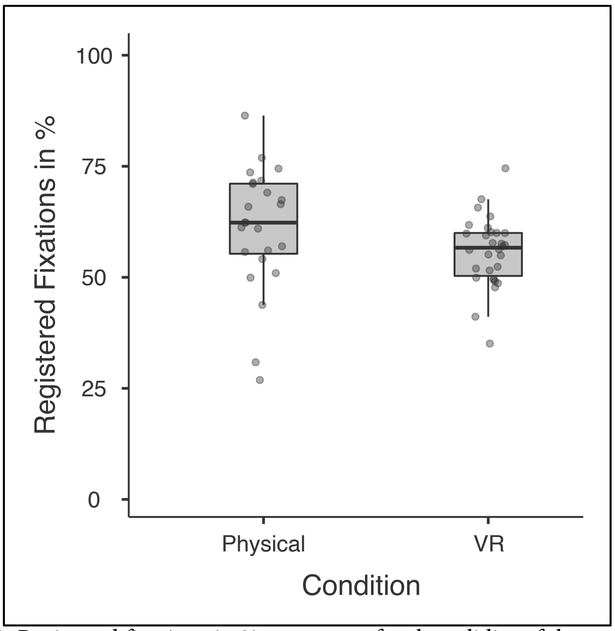

Supplementary Figure S13. Registered fixations in %, as a proxy for the validity of the gaze data: here, the registered fixation in % was simply calculated by the total fixation duration divided by the total recording duration (i.e., total viewing duration). The remaining percentage accounts for all non-fixational metrics such as saccades, as well as any data loss. In both conditions, the registered fixations in percentage ($M_{Physical}$ = 61.1, $SEM_{Physical}$ = ±2.83; M_{VR} = 55.8, SEM_{VR} = ±1.43) showed normal distribution based on the Shapiro-Wilk tests ($W_{Physical}$ = .946, p = .221; W_{VR} = .973, p = .625), and a similarity between the two conditions was assumed based on a comparison using a simple GLM that resulted in a non-significant result ($F_{(1,52)} = 3.19$, $p > .05$, $\eta_p^2 = .058$). The boxplots are based on the distribution of the means from participants, and each individual data point shown as an overlay on top of the boxplots represents a single participant. The categorically separated x-axis denotes two conditions, and the y-axis denotes the registered fixations in percentage. The sample sizes were $N_{Physical}$ = 24, N_{VR} = 30.

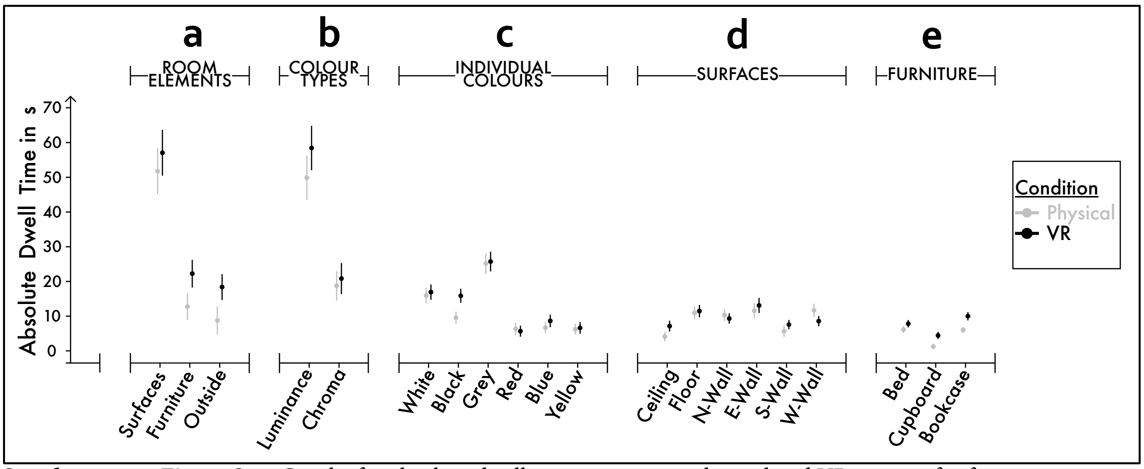

Supplementary Figure S14. Graphs for absolute dwell time comparing physical and VR settings for five comparisons: (a) room elements, (b) colour types, (c) individual colours, (d) surfaces, and (e) furniture. The x-axis shows the levels of individual AOIs, and the y-axis shows absolute dwell time in seconds. Physical and VR conditions were colour-coded as grey and black, respectively. The visualized data were based on means, with whiskers indicating standard errors. The sample sizes were $N_{\text{Physical}} = 24$, $N_{\text{VR}} = 30$.

The analysis method for absolute dwell time (defined as time spent on looking on particular regions irrespective of the size of these regions, namely, cumulative fixation duration per AOI) was the same compared to area-normalized dwell time, using linear mixed-effects models for five comparisons.

Comparison 1 on room elements (a): A significant difference between room elements was found, but no difference was observed between environments or in terms of an interaction: $F_{(2, 46.5)} = 30.605$, $p < .001$; $F_{(1, 30.4)} = 2.503$, $p > .05$; and $F_{(2, 104.3)} = 0.322$, $p > .05$ respectively. Dwell time on surfaces ($M_{Surfaces} = 55.62 \pm 5.79$s) was higher than both furniture ($M_{Furniture} = 18.17 \pm 1.79$s) and outside ($M_{Outside} = 14.26 \pm 1.58$s): $t_{(28.9)} = 7.06$, $p < .001$; and $t_{(28.9)} = 7.73$, $p < .001$, respectively. Overall, in both environments visitors spent most time looking at surfaces of the installation in comparison to furniture elements or outside space.

Comparison 2 on colour types (b): A significant difference between colour types was found, but no difference was observed between environments or in terms of an interaction: $F_{(1, 32.6)} = 72.091$, $p < .001$; $F_{(1, 30.5)} = 0.617$, $p > .05$; and $F_{(1, 53.3)} = 1.635$, $p > .05$, respectively. The dwell time on luminance-only colours ($M_{Luminance} = 54.25 \pm 5.24$s) was higher than chroma-containing colours ($M_{Chroma} = 19.55 \pm 2.09$s). Overall, in both environments visitors spent more time looking at black, grey and white colours in comparison to red, blue, and yellow.

Comparison 3 on individual colours (c): Significant differences between individual colours was found, no difference was observed between environments, and a difference was present in terms of an interaction: $F_{(5, 56.7)} = 17.579$, $p < .001$; $F_{(1, 30.6)} = 0.524$, $p > .05$; and $F_{(5, 233.0)} = 2.451$, $p = .034$; respectively. Visitors spent most time on grey ($M_{Grey} = 25.20 \pm 2.53$s), and least on red ($M_{Red} = 5.78 \pm 3.63$s). When the interaction was broken down to check the relationship between two environments and six colour types, we found little differences for any level of colour: all six post hoc comparisons yielded non-significant results (where all $p > .05$). The interaction was only pronounced, when it was broken down by focusing on the individual colours to check how paired colour differences are affected differently for VR and physical environment: in this approach, some trend changes were visible, such as the dwell time difference between black_{VR} and grey_{VR} was significant ($p = .005$), but the dwell time difference between black_{VR} and $\text{grey}_{\text{Physical}}$ was not significant as ($p > .05$), suggesting that for some colour pairs, the amount of dwell time difference could be dependent on the environment.

Comparison 4 on cube surfaces (d): A significant difference between cube surfaces was found, no difference was observed between environments, and an effect was present in terms of an interaction: $F_{(5, 54.4)} = 9.819$, $p < .001$; $F_{(1, 29.9)} = 0.068$, $p > .05$; and $F_{(5, 231.5)} = 2.653$, $p = .024$, respectively. The dwell time on the ceiling ($M_{Ceiling} = 5.66 \pm 0.89$s) was lowest, and on the east-wall ($M_{East\text{-}Wall} = 12.80 \pm 1.85$s) was highest. Overall, visitors looked approximately five to thirteen seconds at each one out of six faces of the cube. When the interaction was broken down to check how the two types of environment interact with six cube surfaces, all six post hoc comparisons yielded non-significant results (where all $p > .05$). The interaction was only pronounced, when the interaction was broken down to check how levels of cube surfaces have a different effect for VR and physical environment: in this approach, some trend changes were visible, such as the dwell time difference between $\text{south-wall}_{\text{Physical}}$ and $\text{west-wall}_{\text{Physical}}$ was significant ($p = .009$), but the dwell time difference between $\text{south-wall}_{\text{Physical}}$ and $\text{west-wall}_{\text{VR}}$ was not significant as ($p > .05$), suggesting that for some paired cube surfaces, the amount of dwell time difference was dependent on the environment.

Comparison 5 on furniture (e): A significant differences between types of furniture was found, a difference was observed between environments, but no effect was present in terms of an interaction: $F_{(2, 26.1)} = 20.21$, $p < .001$; $F_{(1, 30.2)} = 7.77$, $p = .009$; and $F_{(2, 63.3)} = 1.22$, $p > .05$, respectively. In terms of furniture, the dwell time on cupboard ($M_{Cipboard} = 3.03 \pm 0.64$s) was lower than both bookcase ($M_{Bookcase} = 8.12 \pm 0.91$s) and bed ($M_{Bed} = 7.01 \pm 0.70$s): $t_{(28.2)} = 5.88$, $p < .001$; and $t_{(28.1)} = 4.74$, $p < .001$, respectively. In terms of environments, the dwell time for furniture in VR ($M_{VR} = 7.41 \pm 0.73$s) was longer than physical ($M_{Physical} = 4.36 \pm 0.36$s): $t_{(28.4)} = 2.77$, $p = .010$. Overall, visitors spent more time looking at furniture in VR compared to in the physical environment, and in both conditions the cupboard was the least fixated furniture out of three.

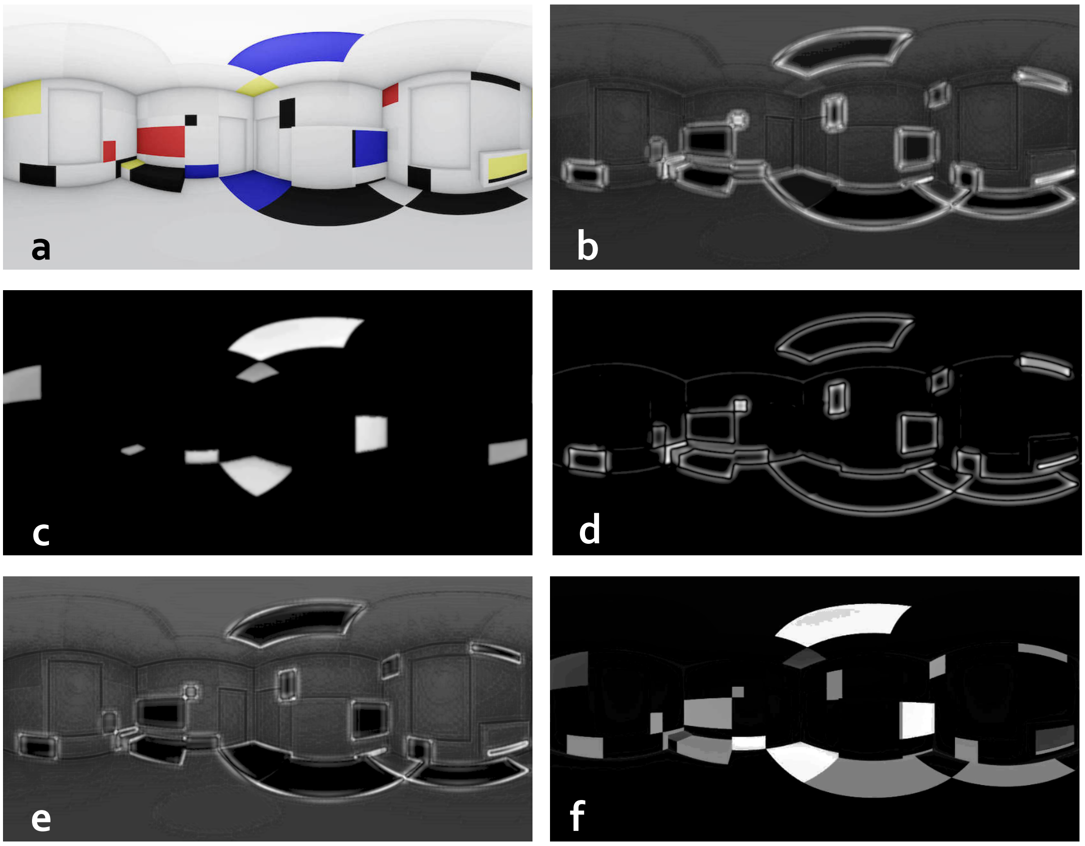

Supplementary Figure S15. (a) Panoramic render of the VR version, (b) a saliency map generated using the Itti algorithm and its (c) colour, (d) intensity, and (e) orientation components; and (f) another saliency map generated using histogram contrast. (b-e) For the Itti algorithm, following the default settings to calculate features using linear centre-surround operations in the RGB colour space, the centre is defined as a pixel at scale $c \in \{2, 3, 4\}$, and the surround is defined as the corresponding pixel at scale $s = c + \delta$ where $\delta \in \{3, 4\}$[3]. (f) The histogram contrast mapping is simply based on mean colour difference to image pixels. Note that, (c) the colour component of the Itti algorithm disregards the black surfaces, whereas (f) a simpler saliency map based on histogram contrast includes black surfaces, which becomes a more suitable saliency map since normalized dwell time was relatively high on both chroma-containing colours and blacks, and relatively low on white and grey surfaces.

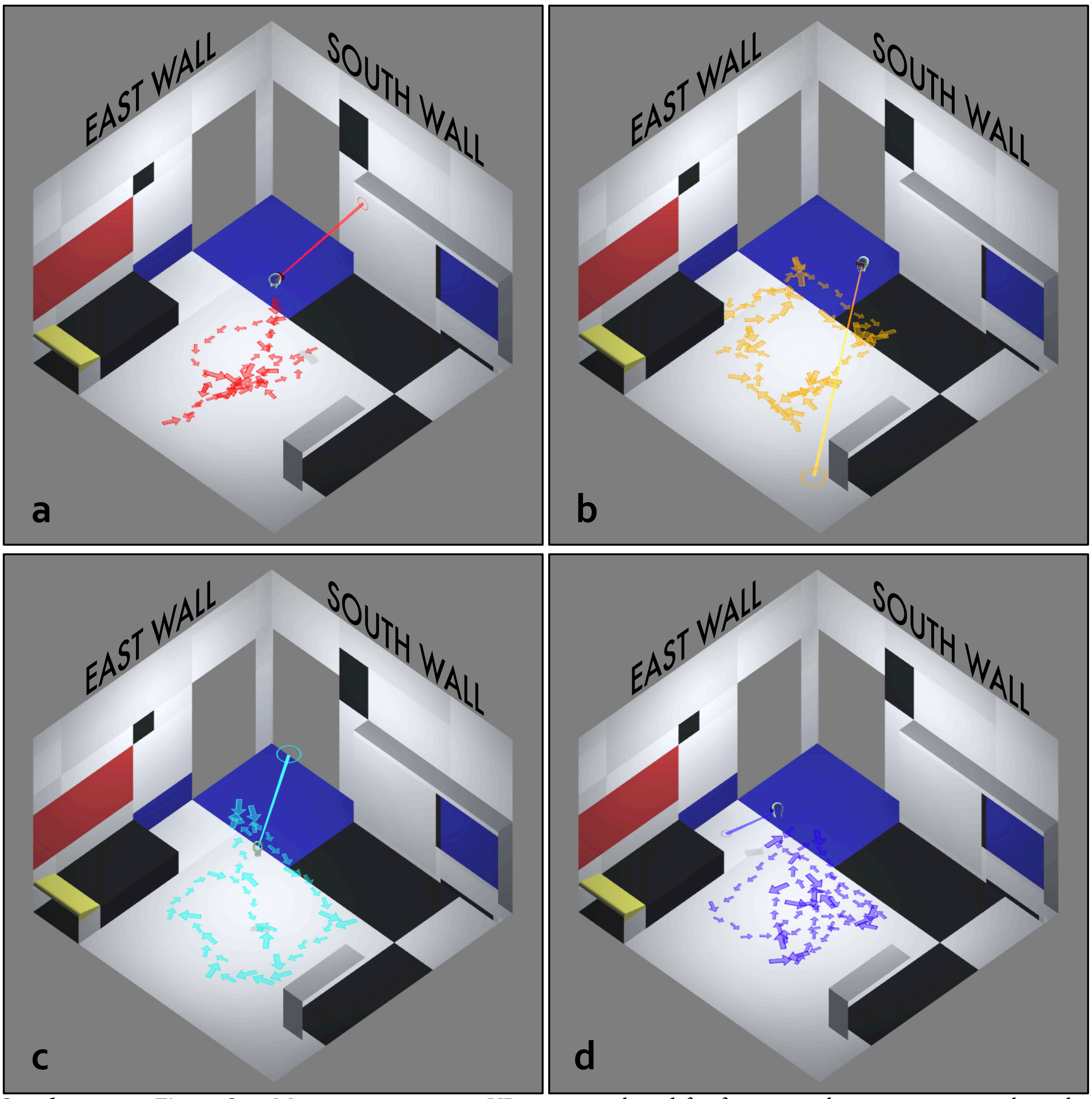

Supplementary Figure S16. Motion trajectories in VR viewing, plotted for four exemplar participants to show the individual differences in terms of general movement within the installation, illustrated by the arrows on the floor.

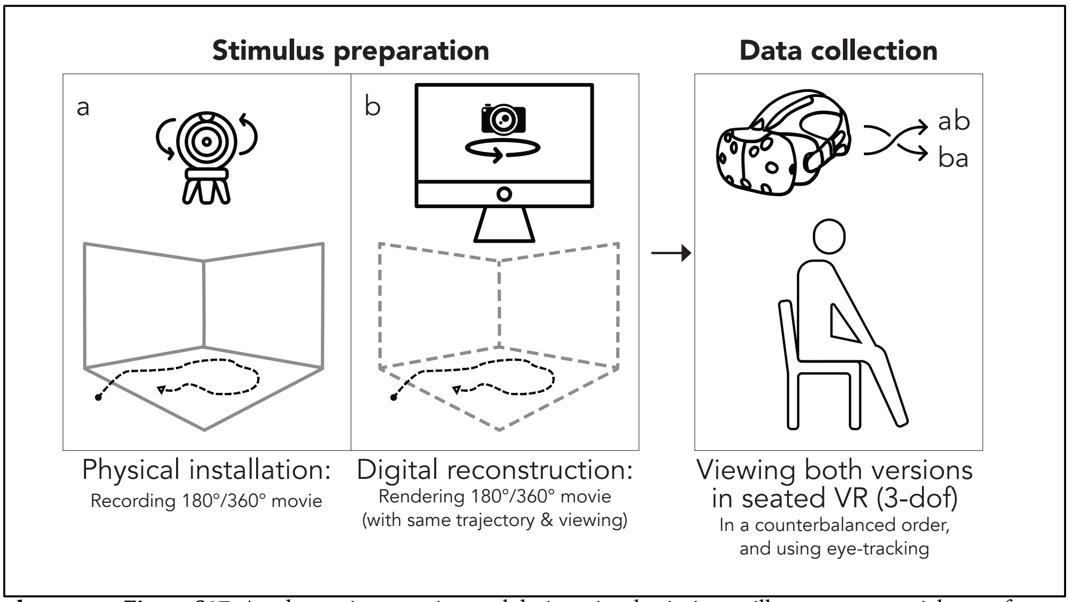

Supplementary Figure S17. An alternative experimental design, simply aiming to illustrate a potential way of equating the movement and the viewing duration between physical and digital settings, whilst still providing partial immersiveness to some extent. Instead of allowing the participant to freely move in two settings without time constraints (as in the case of our presented study); alternatively, the 180° or 360° video footage can be recorded from the physical museum, and using the same trajectory and viewing angles, a virtual replication of a rendered movie can be created. After that, both versions of the videos, basically as a form of "a guided viewing", can be viewed by the participants in a seated-VR as full or semi-spherical movies in a counter-balanced order in the case of a within-subjects experiment, (or these movies can be viewed by two groups of participants, if the design is a between-subjects experiment).

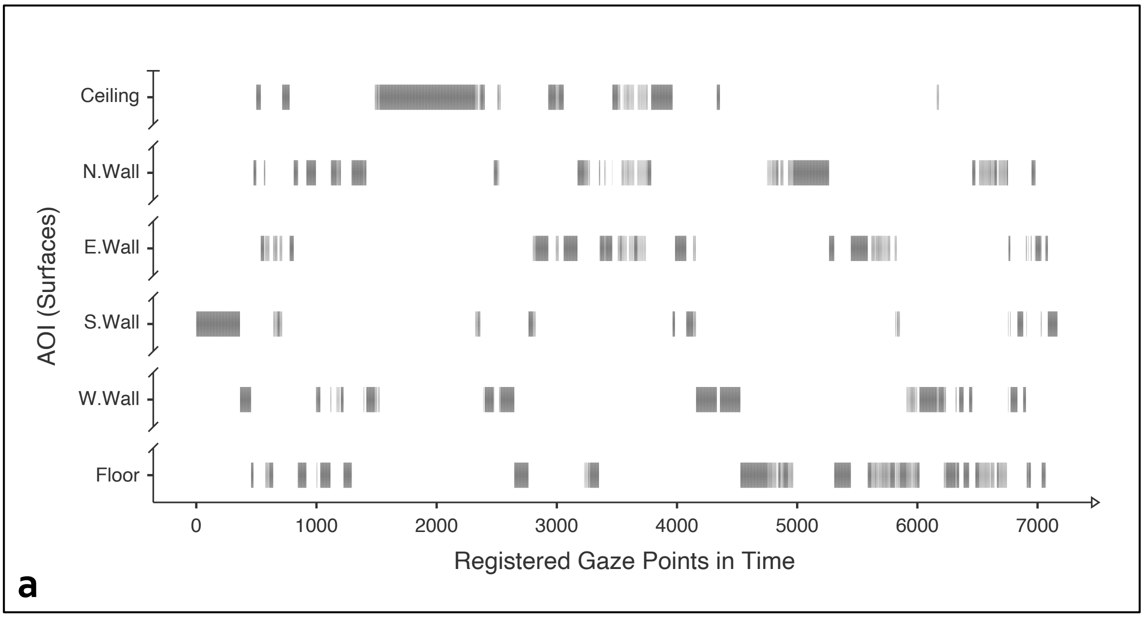

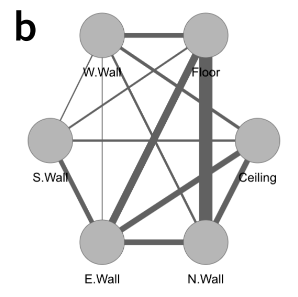

c

	Ceiling	N.Wall	E.Wall	S.Wall	W.Wall	Floor
**Ceiling**	0	.017	.060	.015	.027	0
**N.Wall**	.057	0	.018	0	.016	.146
**E.Wall**	.021	.057	0	.043	0	.077
**S.Wall**	.015	0	.044	0	.004	.011
**W.Wall**	.025	.016	.001	.002	0	.047
**Floor**	0	.149	.076	.012	.044	0

Supplementary Figure S18. (a) The plot is showing the evolution of the registered gaze points (i.e., raw data to be used to classify fixations) over time, here, for a single exemplar participant in the VR condition. The x-axis shows the order of the registered gaze points in time, the lanes on the y-axis show the corresponding AOI (here, out of six cardinal surfaces), and each datapoint displayed as a thin slice on the plot is a single registered gaze point in a temporal order. This temporal illustration, for example, shows that the participant stated by particularly looking on the south wall at start, and after some time, spend significant time viewing the ceiling, etc. (b) A graph-theoretical representation for registered gaze point changes for the same participant (as an approach for the scan path similarity): in the weighted undirected graph, the nodes denote set of AOIs, here illustrated only as six cardinal surfaces, and each edge with a stroke width denotes the amount of gaze point change from one AOI to another, disregarding loops, which can be formed if two or more consecutive gaze points fall on the same AOI. (c) The corresponding adjacency matrix, denoting the amount of registered gaze point change from one AOI to another, reported as a percentage of all fixation data. Note, the amount of change either equates or approximates zero for the surface pairs orthogonal to each other (i.e., ceiling-floor, north wall – south wall, east wall – west wall), since it is unlikely to observe such fixational jumps.

References

1. Salvucci, D. D. & Goldberg, J. H. Identifying fixations and saccades in eye-tracking protocols. in *Proceedings of the symposium on Eye tracking research & applications - ETRA '00* 71–78 (ACM Press, 2000). doi:10.1145/355017.355028.
2. Komogortsev, O. V., Gobert, D. V., Jayarathna, S., Do Hyong Koh & Gowda, S. M. Standardization of automated analyses of oculomotor fixation and saccadic behaviors. *IEEE Trans. Biomed. Eng.* **57**, 2635–2645 (2010).
3. Itti, L., Koch, C. & Niebur, E. A model of saliency-based visual attention for rapid scene analysis. *IEEE Trans. Pattern Anal. Mach. Intell.* **20**, 1254–1259 (1998).
